# A plasmid-encoded genetic switch orchestrates the bacterial virulence-fitness trade-off to drive antibiotic resistance dissemination

**DOI:** 10.1128/mbio.01899-26

**Published:** 2026-09-16

**Authors:** Hongcheng Wei, Wenlan Yang, Tiejun Zhang, Wei Xu, Ke Wu, Linghan Kong, Hao Li, Kailu Wang, Wenjun Yan, Ming Yang, Changyu Zhou, Songhua Li, Zhonghan Li, Changwei Lei, Hongning Wang

**Affiliations:** 1 Laboratory of Bio-Resource and Eco-Environment of Ministry of Education, College of Life Sciences, Sichuan University 12530, Chengdu, Sichuan, China; 2 Animal Disease Prevention and Food Safety Key Laboratory of Sichuan Province, Chengdu, China; 3 School of Laboratory Medicine, Chengdu Medical College 74787, Chengdu, Sichuan, China; 4 Chengdu Academy of Educational Sciences, Chengdu, Sichuan, China; 5 Inner Mongolia Key Laboratory for Molecular Regulation of the Cell, College of Life Sciences, Inner Mongolia University 12576, Hohhot, China; University of Florida College of Dentistry, Gainesville, Florida, USA

**Keywords:** multidrug-resistant plasmids, T1FR, LtrA, virulence-fitness trade-off, antibiotic resistance

## Abstract

The global dissemination of multidrug-resistant plasmids poses a major threat to antimicrobial therapy. While these plasmids are known to carry antibiotic resistance genes, how they balance bacterial fitness and virulence to ensure their own persistence and dissemination remains unclear. Here, we identify a previously unannotated IncFII plasmid-encoded protein, which we name type 1 fimbrial repressor (T1FR). T1FR directly binds the *fimS* phase-variation switch, locking it in the “off” orientation to suppress type 1 fimbria (T1F) biogenesis, thereby attenuating bacterial adhesion, colonization, and pathogenicity. Concurrently, T1FR reprograms central carbon metabolism to offset the fitness cost of plasmid carriage, thereby enhancing plasmid transconjugative efficiency and host competitive fitness. This dual function is dynamically modulated by LtrA-mediated suppression of T1fr transcription, introducing plasticity into the virulence-fitness trade-off. Collectively, the LtrA-T1FR system orchestrates an evolutionary trade-off between plasmid fitness and host pathogenicity. Targeting the T1FR-T1F axis may offer a novel anti-adhesion therapeutic that avoids selection for antibiotic resistance.

**IMPORTANCE:**

Bacterial resistance has become one of the most serious public health issues worldwide. The evolution and spread of drug-resistant plasmids represent one of the significant approaches for bacteria to acquire resistance, and drug-resistant bacteria often have a high mortality rate. However, the association and regulatory mechanism between antibiotic-resistant plasmids and bacterial virulence are currently unclear. The IncFII plasmid is considered an epidemic-resistant plasmid and plays a critical role in the widespread spread of carbapenem antibiotic resistance genes. This study identified a novel protein, T1FR type 1 fimbrial repressor), in IncFII plasmids and explored its regulatory mechanism by inhibiting the generation of host bacterial type 1 fimbriae, thereby weakening bacterial virulence and facilitating stable plasmid transmission. This study provides the scientific basis for understanding the correlation between bacterial resistance and virulence and promotes the scientific prevention and comprehensive management of bacterial infections.

## INTRODUCTION

Bacteria evolve resistance to antibiotics through chromosomal mutations or acquisition of mobile genetic elements (MGEs) such as plasmids (1). Conjugative plasmids are major drivers of this crisis, acting as vectors that disseminate antibiotic resistance genes (ARGs) across bacterial populations and even across species boundaries (2, 3). However, plasmids are not merely passive cargo carriers; they impose fitness costs on their bacterial hosts, including reduced growth rates, metabolic burden, and increased susceptibility to environmental stresses (4, 5). To persist and spread, multidrug-resistant (MDR) plasmids must, therefore, evolve strategies that mitigate these costs while maintaining their ability to confer resistance under antibiotic selection (6, 7). A central but unresolved question is how MDR plasmids balance the trade-off between the fitness burden they impose and the virulence of their bacterial hosts—a question that lies at the heart of understanding the evolutionary success of resistance plasmids.

Among the diverse plasmid families, IncFII plasmids are particularly successful vectors for carbapenemase genes such as *bla*_NDM_ and *bla*_KPC_, which confer resistance to last-line antibiotics (5, 8, 9). These plasmids are characterized by their large size, low copy number, incompatibility with other IncF group plasmids, and global distribution among *Enterobacteriaceae* (8–10). Complete sequencing of epidemic IncFII plasmids has revealed that, beyond carrying ARGs, they encode dozens of hypothetical proteins whose functions remain unknown (11, 12). This observation raises the possibility that some of these unannotated proteins may enable IncFII plasmids to modulate host bacterial physiology—potentially to offset the fitness costs of plasmid carriage or to alter bacterial virulence—thereby facilitating their own dissemination.

Type 1 fimbriae (T1F), a critical virulence determinant in *Enterobacteriaceae*, mediates bacterial adhesion to host surfaces, biofilm formation, and colonization (13–15). In *Escherichia coli*, T1F biogenesis is tightly controlled by phase variation of the *fimS* invertible element, a 314-bp DNA segment containing the promoter for the *fim* operon (16, 17). When *fimS* is in the “on” orientation, the *fim* gene cluster (including structural components [*FimA* polymers], the tip adhesin [*FimH*], and the dedicated secretion system [*FimC/D/F/G* chaperone-usher complex]) is transcribed, leading to T1F assembly; when it inverts to the “off” orientation, transcription is silenced (16–18). This switch is modulated by a well-characterized network of host-encoded regulators, including the histone-like nucleoid structuring protein (H-NS), leucine-responsive regulatory protein (Lrp), and integration host factor (IHF) (16, 19). Notably, some MDR plasmids encode functional orthologs of these regulators, including H-NS-like proteins such as *Shigella flexneri* H-NS-like protein (Sfh), which remodels host chromatin to facilitate plasmid integration (20, 21). These precedents led us to hypothesize that IncFII plasmids may encode previously unknown proteins that directly modulate T1F expression—thereby influencing both bacterial virulence and the fitness consequences of plasmid carriage.

Here, we test this hypothesis and uncover a previously unannotated IncFII plasmid-encoded protein, which we name type 1 fimbrial repressor (T1FR). We demonstrate that T1FR directly binds the *fimS* invertible element and locks it in the “off” orientation, thereby suppressing T1F biogenesis, attenuating bacterial adhesion and colonization, and reducing pathogenicity in both insect and mammalian infection models. Concurrently, T1FR reprograms bacterial central carbon metabolism to alleviate the fitness cost associated with plasmid carriage, promoting both vertical and horizontal transmission of MDR plasmids. Strikingly, the expression of *T1fr* is itself repressed by the plasmid-encoded reverse transcriptase LtrA, which binds the *T1fr* promoter and antagonizes T1FR function. This LtrA-T1FR antagonism creates a dynamic regulatory switch that fine-tunes the trade-off between virulence and fitness. Global genomic analysis reveals that *T1fr*-carrying plasmids are widely distributed across at least seven *Enterobacteriaceae* species, predominantly of the IncFII type, and that the presence or absence of *ltrA* correlates with distinct ARG profiles. Collectively, our findings define the LtrA-T1FR system as an evolutionary strategy by which MDR plasmids orchestrate the virulence-fitness trade-off to ensure their own dissemination. Finally, we propose that targeting the T1FR-T1F axis may offer a novel anti-adhesion therapy that disarms pathogens without selecting for antibiotic resistance, addressing a pressing unmet need in the treatment of *Enterobacteriaceae* infections.

## RESULTS

### The pEC51-encoded LtrA protein maintains host bacterial T1F

**Fig 1:**
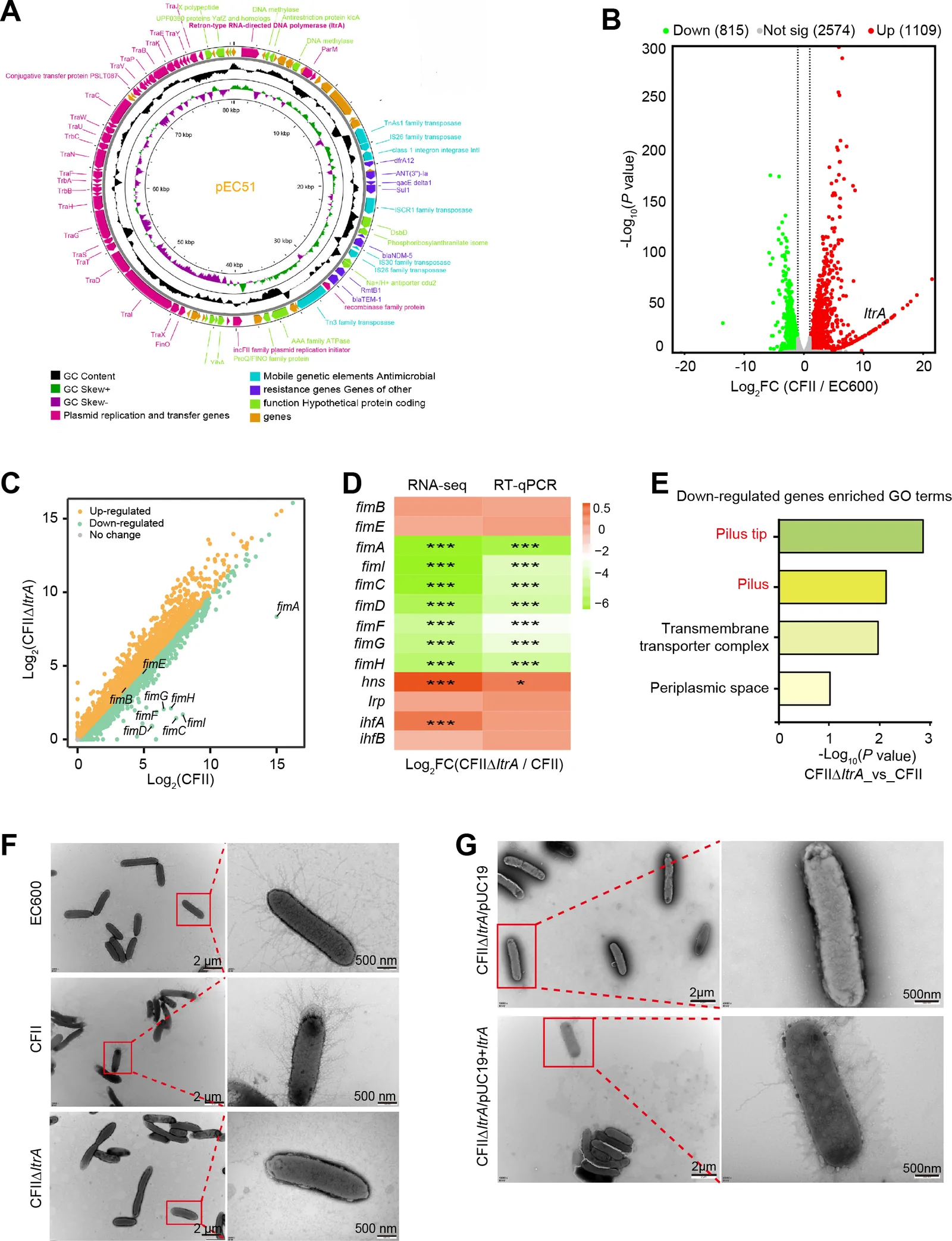
The plasmid-encoded *ltrA* gene maintains T1F biogenesis in host bacteria. (**A**) Genomic landscape of plasmid pEC51 harboring multidrug resistance genes. (**B**) Volcano plot showing the genes differentially expressed in EC600 bacteria harboring the pEC51 plasmid (CFII strain) compared to the wild-type plasmid (EC600). Upregulated genes were marked with red dots; downregulated genes were marked with green dots. (**C**) Volcano plot of genes differentially expressed in EC600 harboring wild-type pEC51 (CFII strain) or *ltrA-*deletion pEC51 plasmid (CFIIΔ*ltrA* strain). Upregulated genes are marked with orange dots; downregulated genes are marked with bright green dots; *fim*-cluster genes are labeled with the gene name. *n* = 3. (**D**) RNA-seq and RT-qPCR analyses of T1F structural gene expression in CFIIΔ*ltrA* versus CFII strains. *n* = 3. “*” indicates *P* < 0.05, “**” indicates *P* < 0.01, and “***” indicates *P* < 0.001, two-tailed Student’s *t* test. (**E**) The GO biological processes of the downregulated genes in the transcriptome data (CFIIΔ*ltrA* vs CFII). Relevant biological functions of fimbriae are labeled in red. (**F**) The surface fimbriae of EC600, CFII, and CFIIΔ*ltrA* visualized by electron microscopy. Scale bar, 2 μm or 500 nm. (**G**) Surface fimbria characteristics of CFIIΔ*ltrA*/pUC19 (vector control) and CFIIΔ*ltrA*/pUC19 + *ltrA* using electron microscopy. Scale bar: 2 μm or 500 nm.

To elucidate the genetic basis of MDR plasmid dissemination, we integrated genomic analysis of the MDR plasmid pEC51 (accession No: OQ230786.1). pEC51 shared a large core genetic backbone with other IncFII plasmids, carrying seven ARGs along with conserved conjugation and replication loci, while the functions of most genes were unknown (Fig. 1A). To assess the impact of pEC51 on host bacteria, we conjugated it into a plasmid-free *E. coli* strain (EC600), generating the transconjugant CFII. RNA-seq analysis revealed that the *ltrA* gene, pEC51-encoding reverse transcriptase, was among the highly upregulated genes in CFII compared to EC600 (Fig. 1B). This observation led us to investigate the functional role of LtrA in plasmid-host adaptation. We, therefore, constructed an *ltrA* deletion mutant (pEC51Δ*ltrA*) and transferred it into EC600, generating CFIIΔ*ltrA*. Both RNA-seq and reverse transcription-quantitative polymerase chain reaction (RT-qPCR) revealed that deletion of *ltrA* led to a significant downregulation of the entire *fim* gene cluster, which encodes the T1F biogenesis machinery (Fig. 1C and D; Fig. S1A). Gene Ontology (GO) analysis further showed that the genes with reduced expression in CFIIΔ*ltrA* were significantly enriched for pilus tip and pilus signaling pathways (Fig. 1E), corroborating the loss of T1F observed by electron microscopy.

Strikingly, these mutants were virtually bald, lacking surface fimbriae, whereas CFII strains displayed abundant fimbriae (Fig. 1F). However, deletion of *ltrA* did not substantially affect the antibiotic resistance of the host bacteria (Table S1). To confirm that the observed phenotypes were specifically attributable to *ltrA* deletion, we performed complementation experiments. Exogenous expression of *ltrA* in CFIIΔ*ltrA* fully restored T1F production (Fig. 1G; Fig. S1B and C). This phenotype was accompanied by attenuated virulence in a *Galleria mellonella* infection model, compromised biofilm formation, and significantly reduced bacterial adhesion (Fig. S1D through S1I). Collectively, these results demonstrate that LtrA is critical for maintaining bacterial T1F, biofilm formation, and full virulence.

### A novel protein T1FR suppresses T1F in bacteria

**Fig 2:**
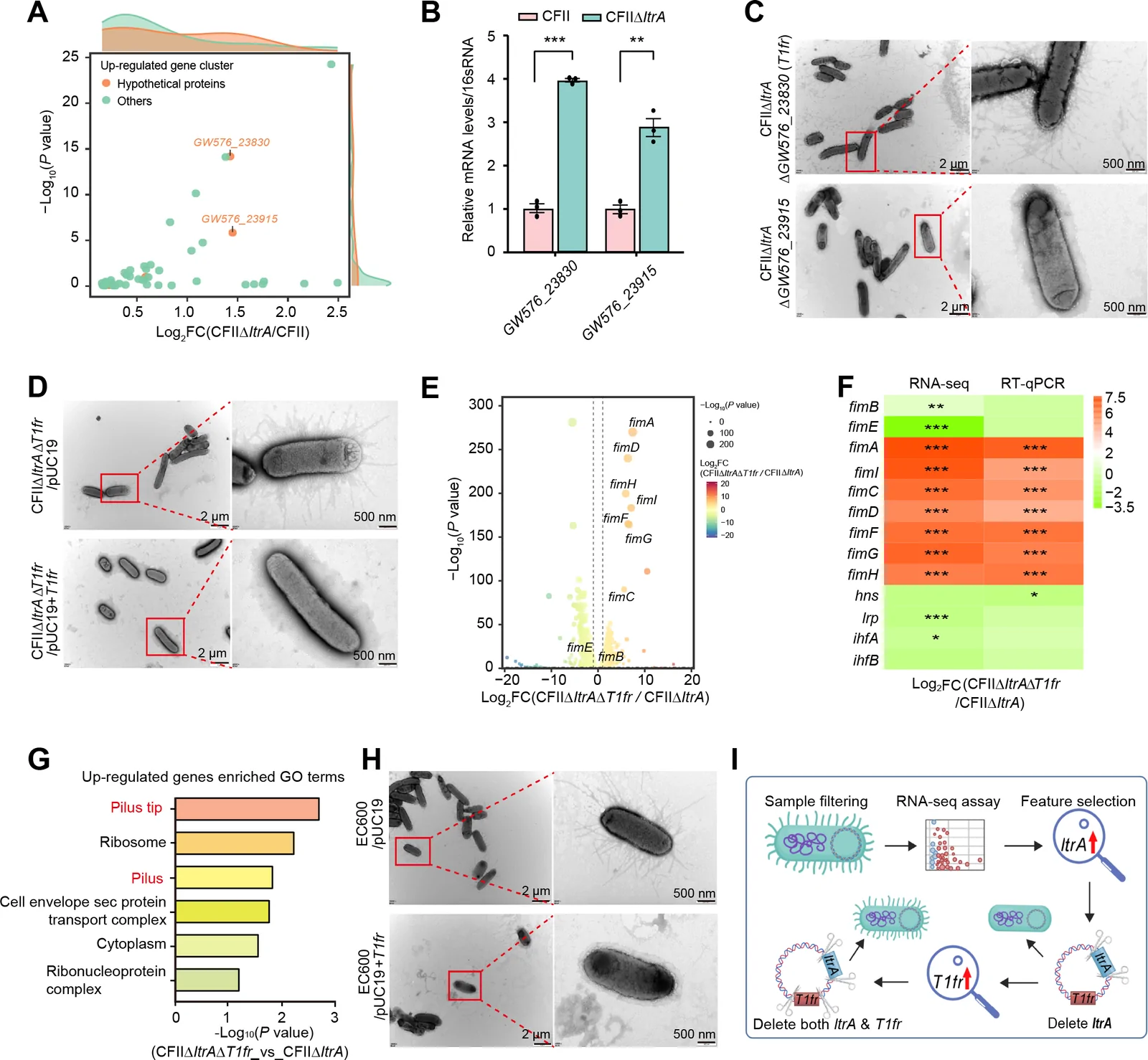
Identification of a novel protein T1FR that inhibits T1F formation in *E. coli*. (**A**) Scatter plot shows the pEC51 plasmid genes that were upregulated in CFIIΔ*ltrA* compared to CFII. Red circles represent genes with established functions. Blue circles denote hypothetical protein-coding genes, including *GW576_23830* and *GW576_23915*. (**B**) Quantitative comparison of *GW576_23830* and *GW576_23915* expression via RT-qPCR analysis in CFIIΔ*ltrA* versus CFII. *n* = 3. * indicates *P* < 0.05, ** indicates *P* < 0.01, and *** indicates *P* < 0.001; two-tailed Student’s *t* test. (**C**) Surface fimbriae of the bacteria after knocking out *T1fr* or *GW576_23915* in the *ltrA*-negative plasmid (CFIIΔ*ltrA*Δ*GW576_23830* (*T1fr*) strain or CFIIΔ*ltrA*Δ*GW576_23915* strain) were detected by electron microscopy. Scale bar, 2 μm or 500 nm. (**D**) Bacterial phenotype of CFIIΔ*ltrA*Δ*T1fr*/pUC19 (vector control) and CFIIΔ*ltrA*Δ*T1fr*/pUC19 + *T1fr* strains detected by electron microscopy. Scale bar: 2 μm or 500 nm. (**E**) Volcano plot of genes differentially expressed between the CFIIΔ*ltrA*Δ*T1fr* strain and CFIIΔ*ltrA* strain. Upregulated genes are marked with red dots; downregulated genes are marked with green dots; *fim*-cluster genes are labeled with the gene name. *n* = 3. (**F**) RNA-seq and RT-qPCR analysis of the expression of *fim*-cluster genes in the EC600 strain carrying both *T1fr* and *ltrA deletion* pEC51 plasmid (CFIIΔ*ltrA*Δ*T1fr* strain). “*” indicates *P* < 0.05, “**” indicates *P* < 0.01, and “***” indicates *P* < 0.001; two-tailed Student’s *t* test. Data represent mean ± SEM of three independent experiments. (**G**) The GO biological processes of the downregulated genes in the transcriptome data (CFIIΔ*ltrA*Δ*T1fr*_*vs*_CFIIΔ*ltrA*). Relevant biological functions of fimbriae are indicated by green underlines. (**H**) The T1F features of both strains EC600/pUC19 and EC600/pUC19 + *T1fr* were identified by using electron microscopy. Scale bar: 2 μm (left) and 500 nm (right). (**I**) A summary of the discovery process that identified the T1FR protein. The *ltrA-*deficient pEC51 plasmid was expressed in EC600, and RNA-seq analysis was performed. *fim* family genes were significantly downregulated, and the T1F of EC600 was not detectable. Further analysis revealed that a hypothetical protein named T1FR encoded by the pEC51 plasmid could inhibit the production of T1F in *E. coli*.

To explore the regulatory mechanism of the pEC51Δ*ltrA* plasmid-mediated bacterial T1F loss, we identified plasmid genes demonstrating significantly upregulated expression in CFIIΔ*ltrA* versus CFII strains (Fig. 2A). Notably, none of the previously characterized T1F regulators were identified among the upregulated genes. Instead, two genes encoding hypothetical proteins (*GW576_23830* and *GW576_23915*) showed significantly increased expression in CFIIΔ*ltrA* (Fig. 2A and B). This observation led us to hypothesize that one or both of these unannotated genes might function as novel T1F repressors.

To test this hypothesis, we individually deleted *GW576_23830* or *GW576_23915* in the CFIIΔ*ltrA* background and examined fimbria production by electron microscopy. Interestingly, deletion of *GW576_23830* fully restored fimbriae in CFIIΔ*ltrA*, whereas deletion of *GW576_23915* had no effect (Fig. 2C). Complementation of *GW576_23830* in the double-knockout strain (CFIIΔ*ltrA*Δ*GW576_23830*/pUC19 +*GW576_23830*) again suppressed fimbria production, confirming that *GW576_23830* encodes a functional T1F repressor (Fig. 2D; Fig. S2A and B). Based on this function, we designated the protein encoded by GW576_23830 as T1FR and its corresponding gene as *T1fr*.

To determine whether T1FR represses T1F at the transcriptional level, we compared the transcriptomes of CFIIΔ*ltrA*Δ*T1fr* and CFIIΔ*ltrA* strains. Both RNA-seq and RT-qPCR revealed that deletion of *T1fr* significantly upregulated the entire *fim* gene cluster (Fig. 2E and F; Fig. S2C). Consistently, GO analysis showed that the genes with increased expression in the absence of *T1fr* were significantly enriched for pilus tip and pilus signaling pathways (Fig. 2G). Functionally, *T1fr* deletion also enhanced biofilm formation (Fig. S2D through G). To further validate T1FR’s repressive activity, we exogenously expressed *T1fr* in wild-type EC600. As expected, EC600/pUC19 + *T1fr* strains lost surface fimbriae, confirming that T1FR alone is sufficient to suppress T1F production (Fig. 2H).

Collectively, these results demonstrate that the IncFII plasmid-encoded T1FR functions as a transcriptional repressor of the *fim* gene cluster, thereby suppressing T1F biogenesis. Notably, T1FR expression is itself negatively regulated by LtrA (Fig. 2I).

### LtrA directly binds the *T1fr* promoter to mediate transcriptional repression

To further investigate the regulatory mechanism underlying LtrA-mediated transcriptional repression of *T1fr*, we evaluated the promoter of the plasmid-encoded *T1fr* gene (22, 23) and predicted its interaction with LtrA (24, 25). As shown in the sequence analysis diagram, the thymine (T) at 29 nucleotides (nt) upstream of the start codon of the *T1fr* gene was predicted to be the transcription start site (Fig. S3A). We predicted that the LtrA-binding region at the *T1fr* gene promoter sequence is 5′-CTTTTATAG-3′ located between −46 and −38 nt upstream of the start codon of *T1fr* (Fig. S3A and B). Further protein and DNA molecular docking analysis confirmed this binding site overlapped with the −10 box of the *T1fr* gene promoter (Fig. S3C). To confirm this prediction, LtrA protein was expressed and purified for electrophoretic mobility shift assays (EMSAs) (Fig. S3D and F). The EMSA assays revealed that the LtrA protein bound to the *T1fr* promoter but not to the *T1fr*-Mut promoter (deficient in the predicted LtrA-binding region) (Fig. S3G and H). Oligo pull-down assays confirmed specific binding of the purified LtrA to the wild-type *T1fr* promoter (Fig. S3I and J). Collectively, these results supported the initial prediction that the −10 box of the *T1fr* promoter serves as an important binding region for LtrA.

### T1FR regulates the *fim* gene cluster expression through modulating the *fimS* orientation in bacteria

**Fig 3:**
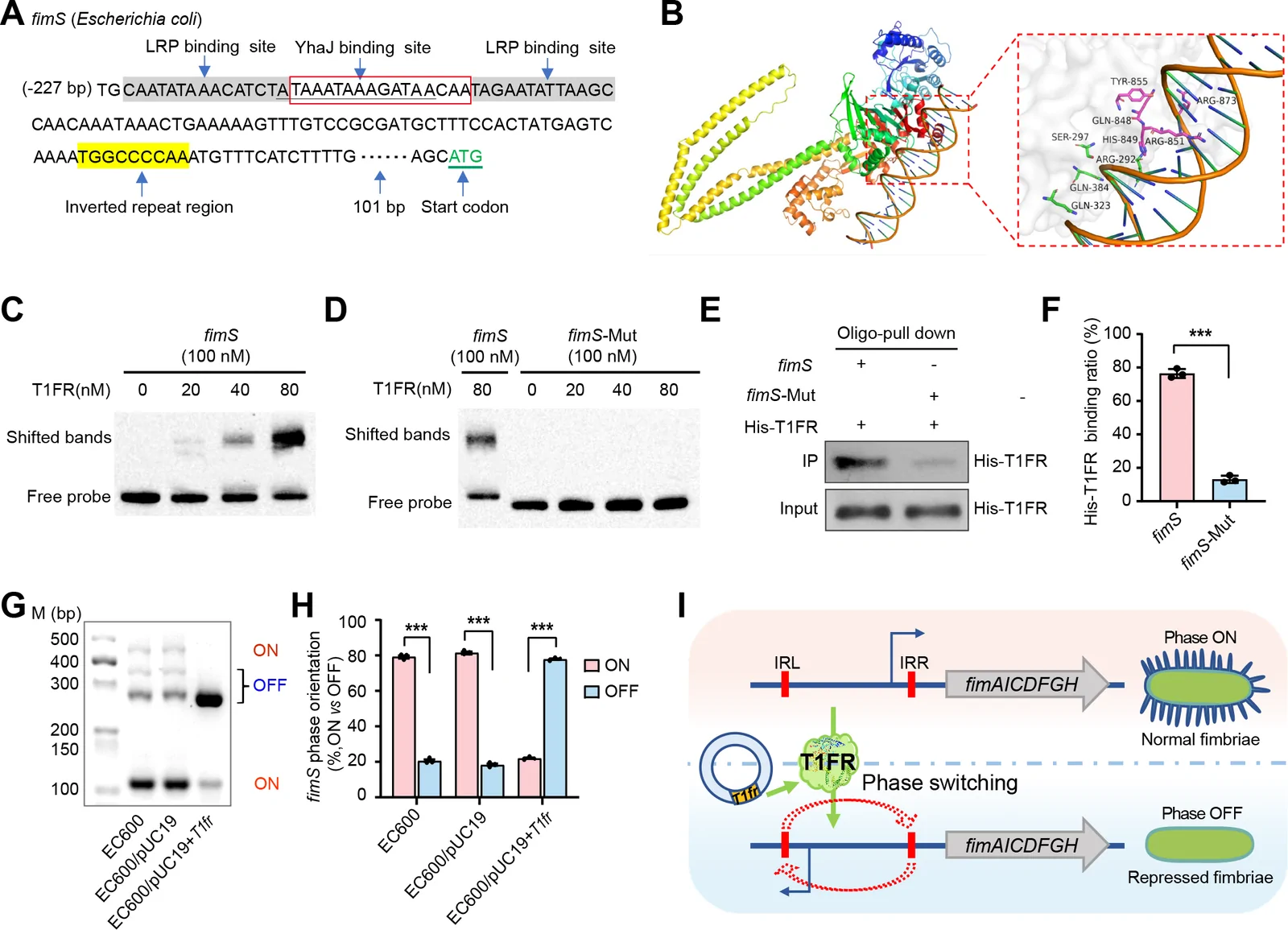
T1FR acts by binding to *fimS*, thereby directing the phase switch to the “off” orientation. (**A**) Sequence organization of the *fimS* regulatory element. Gray shading indicates the LRP-binding site. Yellow shading indicates the inverted repeat region. The underlined region represents the YhaJ-binding site. The red box indicates the putative T1FR-binding region. Green letters indicate the start codon. (**B**) Molecular docking simulation of T1FR-*fimS* interactions. Predicted binding conformation showing T1FR (surface representation) bound to *fimS* DNA (orange cartoon). (**C**) The binding efficiency of T1FR purified proteins with *fimS* was measured by EMSA. (**D**) The binding efficiency of T1FR purified proteins with *fimS*-Mut promoter (putative T1FR-binding region deleted) was detected by EMSA. (**E**) T1FR protein was pulled down by *fimS* oligos. The expression of T1FR in the *fimS* or *fimS*-Mut groups was confirmed by Western blotting using the anti-His antibody. (**F**) Quantification of the band gray signals in the Western blot of panel **E**. * indicates *P* < 0.05, ** indicates *P* < 0.01, and *** indicates *P* < 0.001; two-tailed Student’s *t* test. Data represent mean ± SEM of three independent experiments. (**G**) *fimS* orientation was analyzed in EC600, EC600/pUC19, or EC600/pUC19 + *T1fr* strains. PCR-based assay showed *fimS* orientation states: top/bottom bands (“on” orientation); middle bands (“off” orientation). (**H**) Proportional quantification of the *fimS* orientation in EC600, EC600/pUC19, or EC600/pUC19 + *T1fr* strain corresponding to panel **G**. *** indicates *P* < 0.001; two-tailed Student’s *t* test. (**I**) The regulatory mechanism model of T1FR-mediated inhibition of *fim* gene cluster expression in *E. coli*. The T1FR protein bound to *fimS* and locked its orientation in the “off” state, thereby inhibiting the expression of *fim* genes within the cluster.

Given that T1FR expression is negatively regulated by LtrA, we, therefore, investigated how T1FR itself controls T1F biogenesis. *fimS*, a phase-variable invertible element harboring the *fimA* promoter, serves as the master genetic switch controlling the transcription of the entire *fim* gene cluster in *E. coli* (26). Sequence analysis of the *fimS* element revealed a putative T1FR-binding motif (5′-TAAATAAAGATAACAA-3′, positions −218 to −203 nt relative to the start codon) (25, 27–29) (Fig. 3A and B). Notably, this element is intercalated between two Lrp-binding sites on the *fimS* operator and shares a 13-nt overlap with the microbial transcription factor YhaJ-binding sequence (30, 31). This result is consistent with the corresponding molecular docking analysis results (Fig. 3B; Fig. S4A). Both oligo pull-down and EMSA assays demonstrated that T1FR protein bound to the *fimS* promoter but not to the *fimS*-Mut (deficient for the predicted T1FR-binding motif) (Fig. 3C through F; Fig. S4B through D).

The gene expression of the *fim* gene cluster is regulated by the orientation of the *fimS* invertible element: transcriptional activation requires *fimS* to be in the “on” orientation, whereas switching to the “off” orientation terminates *fim* expression and abolishes T1F assembly (26, 29). Compared with the EC600 strain, the *fimS* switch in the *ltrA*-deletion strain (CFIIΔ*ltrA*) was biased toward the “off” orientation (Fig. S5A and B), but complementation of *ltrA* in CFIIΔ*ltrA* strains (CFIIΔ*ltrA*/pUC19 +*ltrA*) turns to the “on” orientation (Fig. S5C and D). Meanwhile, deletion of *T1fr* in CFIIΔ*ltrA* strains (CFIIΔ*ltrA*Δ*T1fr*) shifted the *fimS* orientation toward “on” (Fig. S5E and F), but rescuing *T1fr* in CFIIΔ*ltrA*Δ*T1fr strains* (CFIIΔ*ltrA*Δ*T1fr*/pUC19 *+T1fr*) shifts it back to the “off” orientation (Fig. S5G and H). Moreover, exogenously expressed T1FR protein in EC600 strains also directly led to *fimS* leaning toward the “off” orientation (Fig. 3G and H). Taken together, the above results suggest that T1FR-regulated *fimS* switch orientation plays a pivotal role in mediating the expression of the *fim* gene cluster that encodes bacterial fimbriae (Fig. 3I).

### T1FR attenuates AIEC virulence and pathogenicity

**Fig 4:**
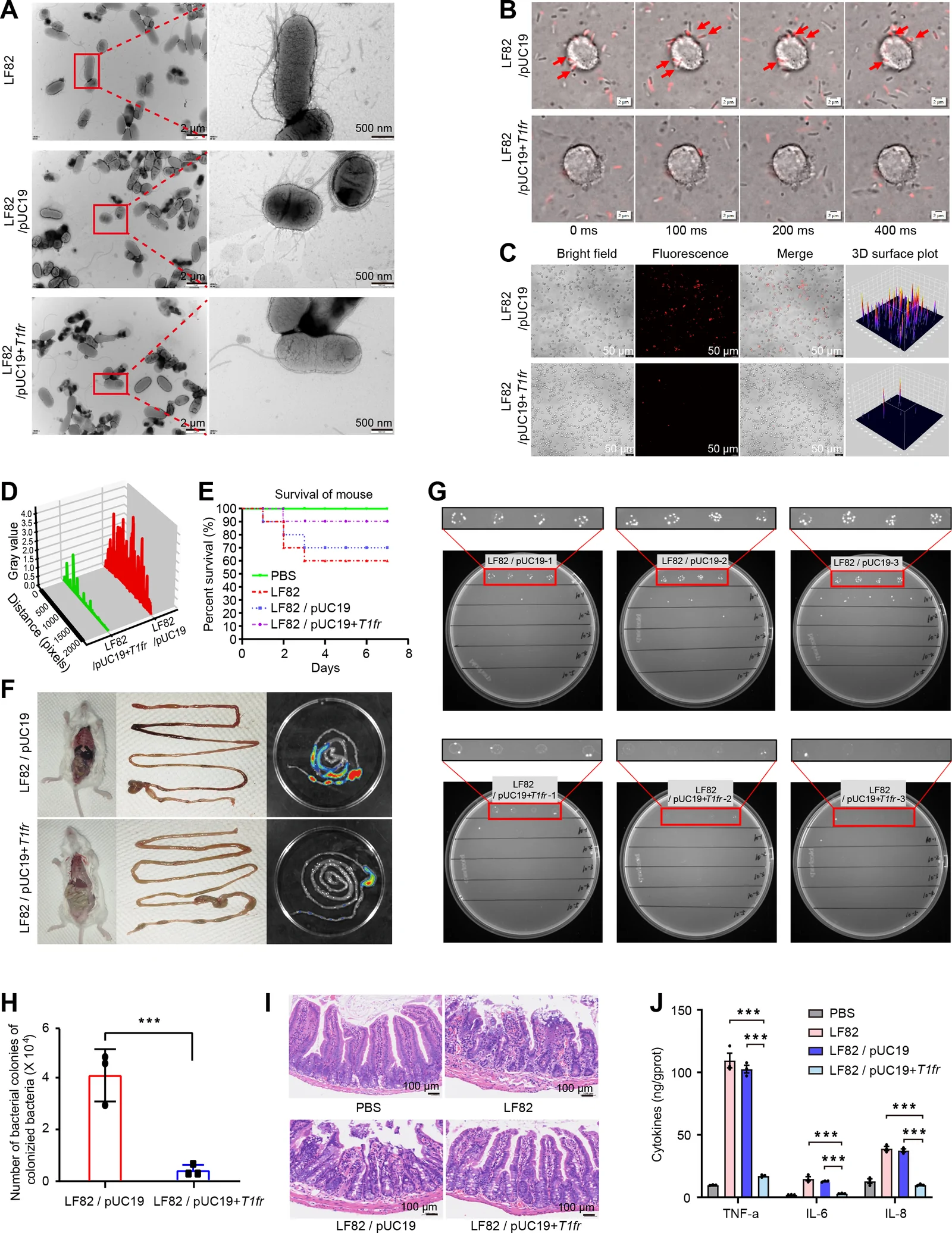
T1FR impairs virulence of AIEC by inhibiting T1F. (**A**) T1F characteristics of LF82 strains carrying the *T1fr*-containing pUC19 plasmid (LF82/pUC19 + *T1fr* strain) or empty pUC19 vector (LF82/pUC19 strain) were visualized by electron microscopy. Scale bar: 2 μm (left) and 500 nm (right). (**B**) Tracking the adhesion process between LF82/pUC19 + *T1fr* or LF82/pUC19 (carrier vector control) strains and RAW264.7 cells using confocal laser scanning microscopy. The bacteria were labeled with red fluorescence. Scale bar: 2 μm. (**C**) The adhesion capacity of LF82/pUC19 + *T1fr* and LF82/pUC19 (vector control) strains onto RAW264.7 cells was detected by cell fluorescence assays. The bacteria were labeled with mCherry. Scale bar: 50 μm. (**D**) Quantitative analysis of adhesion ability for LF82/pUC19 + *T1fr* or LF82/pUC19 strains onto RAW264.7 cells using ImageJ (Fuji) software to characterize the signal of mCherry in Fig. 2C. Both LF82/pUC19 + *T1fr* and LF82/pUC19 strains expressed mCherry signaling. (**E**) Survival curves of mice infected with wild-type LF82, LF82/pUC19, or LF82/pUC19 + *T1fr* strains; phosphate-buffered saline (PBS) was used as blank control, *n* = 10 mice per group. (**F**) The intestinal pathological phenotypes of mice infected with LF82/pUC19 or LF82/pUC19 + *T1fr* strains, and imaging analysis of colonization by mCherry-expressing bacteria (red signal). (**G**) Colony counts to detect the colonization of LF82/pUC19 and LF82/pUC19 + *T1fr* strains in mouse intestines. All data are representative of three independent experiments. (**H**) Quantitative statistics of the colonization of LF82/pUC19 and LF82/pUC19 + *T1fr* strains in mouse intestines. *n* = 3, *** indicates *P* < 0.001, two-tailed Student’s *t* test. (**I**) Histopathological analysis of mouse intestines infected with LF82, LF82/pUC19, or LF82/pUC19 + *T1fr* strains; PBS was used as blank control. Hematoxylin and eosin (H&E) staining is shown above. Scale bar = 100 µm. (**J**) Cytokine expression profiles in intestinal tissues. ELISA quantification of the expression of TNF-α, IL-6, and IL-8 in mice infected with LF82, LF82/pUC19, or LF82/pUC19 + *T1fr* strains; PBS was used as blank control. *n* = 3, *** indicates *P* < 0.001, two-tailed Student’s *t* test.

To further functionally characterize *T1fr*, we complemented the adherent-invasive *E. coli* (AIEC) strain LF82 with a pUC19-based *T1fr* expression construct (LF82/pUC19 + *T1fr* strains). LF82 is a prototypical AIEC strain that clinically mediates intestinal adhesion via type 1 fimbriae, forms biofilms, and triggers Crohn’s enteritis (14, 15). Notably, heterologous expression of T1FR abolished T1F production in LF82, establishing its conserved repressive function in a pathogenic *E. coli* background (Fig. 4A; Fig. S6A through C). Live confocal laser scanning microscopy showed that the LF82/pUC19 + *T1fr* strains additionally displayed attenuated adhesion to RAW264.7 cells, which is consistent with the cell fluorescence assay results of RAW264.7 cells and HIEC-6 cells (Fig. 4B through D; Fig. S6D and E). Concurrently, heterologous expression of *T1fr* in LF82 strains significantly attenuated its virulence, as evidenced by decreased mortality rates in *G. mellonella* infection bioassays (Fig. S6F). The LF82/pUC19 + *T1fr* strains’ biofilm-forming capacity was significantly restricted (Fig. S6G through J). Taken together, these findings demonstrate that the T1FR protein attenuates bacterial virulence by suppressing T1F biogenesis in AIEC strains.

T1F plays a pivotal role in bacterial pathogenesis as it is a critical virulence determinant during the adhesion-invasion phase of AIEC-mediated inflammatory bowel disease (15). To delineate the physiological role of T1FR in AIEC pathogenesis, we established a murine infection model via inoculation of LF82 strains carrying either the pUC19 or pUC19 +*T1fr* plasmid. Mice infected with LF82/pUC19 +*T1fr* strains exhibited higher survival rates compared to LF82/pUC19 controls (Fig. 4E) and reduced intestinal bacterial colonization (Fig. 4F through H). Moreover, intestinal villi in mice infected with LF82/pUC19 +*T1fr* showed virtually no lesions, whereas both wild-type and empty vector-carrying LF82 strains induced intestinal inflammation, mucin depletion, and a reduction in villi numbers and length in mice (Fig. 4I). Levels of the proinflammatory cytokines TNF-α, IL-6, and IL-8 were significantly lower in mice infected with LF82/pUC19 +*T1fr* strains compared to those infected with non-*T1fr*-carrying LF82 strains (Fig. 4J). Meanwhile, while T1FR expression dampened host inflammatory responses, it had no detectable effect on gut microbiota α-diversity or β-diversity (Fig. S7). In summary, these findings demonstrated that T1FR suppresses AIEC intestinal colonization by targeted repression of T1F-mediated adhesion, thereby significantly attenuating AIEC-induced intestinal damage.

### T1FR alleviates plasmid fitness cost to promote ARG dissemination

**Fig 5:**
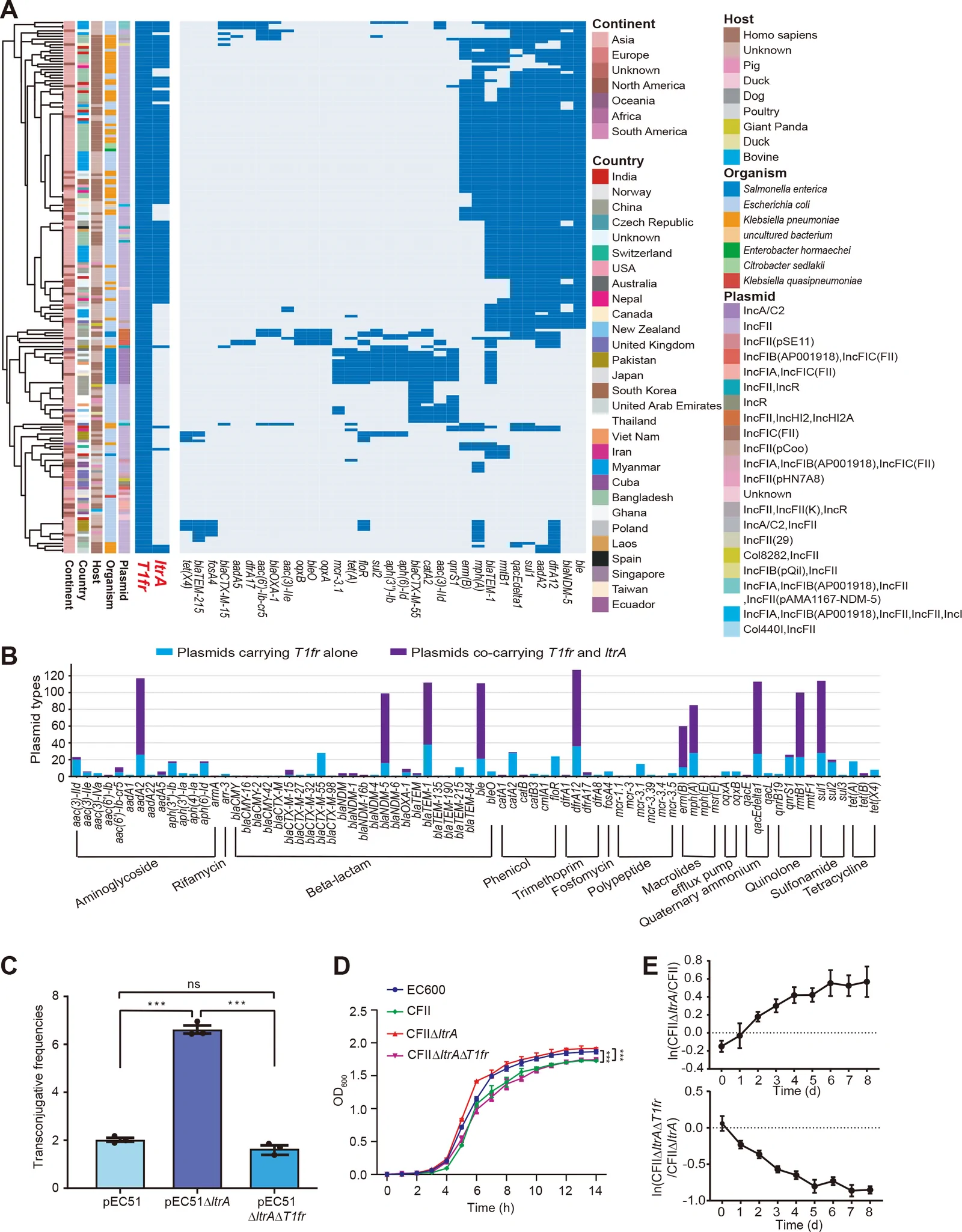
*T1fr* drives the global dissemination of resistance plasmids in *Enterobacteriaceae* by alleviating the associated fitness burden. (**A**) The bootstrapped phylogenomic tree was constructed with *T1fr-*carrying plasmids. These plasmids were obtained from the GenBank database or archived in our laboratory. The evolutionary relationship of different plasmid types (left). The plasmid information of carrying species, origin country and continent, host bacteria, whether carrying *T1fr* and *ltrA*, and ARGs (right). The specific information was distinguished by different colors in the figure legend. (**B**) Antibiotic resistance gene profiles of plasmids encoding *T1fr* alone versus those co-carrying *T1fr* and *ltrA*. (**C**) Transconjugative frequencies of pEC51, pEC51Δ*ltrA,* and pEC51Δ*ltrA*Δ*T1fr* were quantified using a standardized filter mating assay. Donor (plasmid-carrying) and recipient strains were mixed at a 1:1 ratio, incubated for 12 h at 37°C, and plated on selective media to determine transconjugant formation. *n* = 3; “***” indicates *P* < 0.001. (**D**) The growth curves of *E. coli* with or without normal *T1fr* gene expression. *n* = 3. (**E**) Competitive fitness of *E. coli* with or without the *T1fr* gene. Population dynamics of the CFIIΔ*ltrA* strain versus the CFII strain (upper panel) and the Δ*ltrA*Δ*T1fr* strain versus the CFIIΔ*ltrA* strain (lower panel) during the 8-day co-culture (mean ± SEM, *n* = 3).

Global genomic analysis revealed that *T1fr* is widely distributed across at least seven *Enterobacteriaceae* species and multiple continents (Fig. 5A; Table S2). *T1fr* exhibits a strong predilection for IncFII-type plasmids, which account for 78.8% of all 213 *T1fr*-carrying plasmids identified in the GenBank database (Fig. 5A; Fig. S8A). Among 96 plasmid types harboring both *T1fr* and *ltrA*, 91.5% belong to the IncFII replicon type (Fig. S8B and C). The vast majority (94.8%) of *T1fr*-positive plasmids carry ARGs, with 93.6% containing three or more resistance genes (Fig. 5A; Fig. S8D). Notably, IncA/C2 plasmids carrying *T1fr* were exclusively found in *S. enterica* and consistently lacked *ltrA* (Fig. 5A). Host range analysis showed that 85.7% of *K. pneumoniae* and 52.5% of *E. coli* carried *T1fr*-positive plasmids, with distinct ARG profiles depending on *ltrA* co-carriage (Fig. 5B; Table S2).

We next investigated how T1FR promotes plasmid dissemination. Plasmid stability assays over ~600 generations showed that pEC51, pEC51Δ*ltrA*, and pEC51Δ*ltrA*Δ*T1fr* were all stably maintained (Fig. S9A). However, pEC51Δ*ltrA* exhibited significantly higher conjugative transfer frequencies than pEC51 or pEC51Δ*ltrA*Δ*T1fr* (Fig. 5C), indicating that T1FR enhances horizontal transfer. Consistent with a role in fitness compensation, strains carrying *T1fr* alone (CFIIΔ*ltrA*) grew faster than those carrying the full plasmid (CFII) or lacking both genes (CFIIΔ*ltrA*Δ*T1fr*) (Fig. 5D, Fig. S9B). In competitive growth assays over 8 days, CFIIΔ*ltrA* outcompeted both CFII and CFIIΔ*ltrA*Δ*T1fr* (Fig. 5E). Similar results were observed in AIEC (Fig. S9C and 9D).

Untargeted metabolomics coupled with transcriptomic profiling revealed that, compared to the CFIIΔ*ltrA*Δ*T1fr* and CFII strains, the expression of *T1fr* in the CFIIΔ*ltrA* background significantly upregulated genes in four core metabolic pathways—β-alanine metabolism, ascorbate/aldarate metabolism, galactose metabolism, and the pentose phosphate pathway (Rich factor >2, Fig. S10A and B)—all of which are central to energy homeostasis. Conversely, pathways involved in glycerolipid and sulfur metabolism, porphyrin synthesis, and peptidoglycan biosynthesis were repressed (Fig. S10C and D). Pathway enrichment analysis confirmed the pervasive impact on metabolism: 15 of the top 25 enriched pathways in the CFIIΔ*ltrA*Δ*T1fr* versus CFIIΔ*ltrA* comparison, and 14 in the CFIIΔ*ltrA* versus CFII comparison, were metabolism-related (Fig. S10E through H). Together, these findings demonstrate that *T1fr* promotes central carbon and energy metabolism, thereby providing a metabolic basis for its fitness-compensatory function.

## DISCUSSION

MDR plasmids provide critical survival advantages to their bacterial host under antibiotic selection pressure. However, these plasmids frequently impose significant fitness burdens on the host bacteria, including metabolic reprogramming, virulence modulation, and alterations to the growth phenotype (32, 33). Among these, IncF-type plasmids, particularly the clinically prevalent IncFII subtype, have been associated with both virulence modulation and the emergence of MDR “superbugs” harboring the *bla*_NDM_ gene that encodes metallo-β-lactamase and other resistance determinants, which collectively contribute to significantly elevated mortality rates attributed to clinical infections (9, 34, 35). However, the complex interplay of trade-offs and synergies among bacterial virulence, drug resistance, and growth performance remains poorly elucidated. Here, we characterized T1FR, an unannotated protein encoded by IncFII plasmids that functions as a critical regulator balancing virulence and fitness costs during the dissemination of antibiotic resistance plasmids in *Enterobacteriaceae*. Functional analyses in *E. coli* revealed that T1FR strongly repressed T1F biogenesis, which reduced bacterial adhesion to eukaryotic cells or tissues, while alleviating the bacterial fitness cost associated with plasmid carriage and promoting plasmid transconjugative frequencies. Thus, the T1FR protein represents a unique adaptive trade-off mechanism employed by MDR plasmids to balance bacterial fitness and virulence.

T1F expression in *E. coli* is controlled by phase variation of the *fimS* invertible element, which is directly coordinated by the site-specific recombinases FimB and FimE (19, 36). The regulatory landscape of T1F phase variation constitutes a complex hierarchical network (37): the transcription factors H-NS, Lrp, and IHF modulate *fimS* orientation by governing recombinase expression or DNA topology (36). For instance, Lrp binds to three conserved sites within *fimS*, inducing DNA bending that biases the switch toward the “on” state for T1F production, while H-NS represses *fimB*/*E* transcription to globally suppress phase variation (31, 36, 38). Previous studies have indicated that loss of T1F in *E. coli* predominantly results from *fimS* inversion to the “off” orientation (19). Here, we demonstrate that T1FR directly interacts with *fimS* DNA at a locus situated between two Lrp-binding motifs (Fig. 3B). This binding mode contrasts with YhaJ, which enhances T1F production by stabilizing the Lrp-*fimS* complex to promote “on” switching (31). Mechanistically, our studies have suggested that T1FR represses T1F biogenesis by binding to *fimS*, which may competitively disrupt Lrp binding and eliminate the bending required for Lrp-mediated phase switching to the “on” orientation, ultimately silencing the *fim* gene cluster. Collectively, by defining the mechanism of T1FR-mediated virulence suppression, our work establishes a framework for targeting bacterial virulence—a promising therapeutic strategy that curbs infection without promoting antibiotic resistance.

T1F is a critical virulence determinant of *E. coli* LF82 (a Crohn’s disease-associated *E. coli* strain) for intestinal colonization, where T1F drives intestinal infection through epithelial adhesion (14, 15). Normal healthy intestinal epithelial cells (IECs) and macrophages exhibit resistance to lipopolysaccharide (LPS)-induced inflammation due to the absence of Toll-like receptor 4 (TLR4)-accessory proteins CD14 or myeloid differentiation factor 2 (MD2) (39). Notably, TLR4 expressed on these CD14-/MD2-deficient cells remains sensitive to bacterial T1F, with their interaction triggering inflammation through induction of TNFα, IL-6, and IL-8 in the luminal epithelium (40, 41). Effective treatment of intestinal diseases has been severely hindered by the ever-increasing prevalence of antibiotic resistance in pathogenic bacteria (14, 42). Broad-spectrum antibiotics eradicate pathogens and also deplete beneficial gut microbiota, exacerbating dysbiosis and creating niches for resistant strain proliferation (41, 43). Hence, targeting the T1F adhesin has emerged as a promising anti-virulence strategy as its inhibition disrupts AIEC colonization and pathogenicity *in vivo* (41, 44). At present, there are few reports on biological factors that disrupt bacterial fimbria-mediated adhesion, thereby inhibiting biofilm formation and pathogenicity. Our study demonstrates that T1FR expression reduces the occurrence of LF82-induced enteritis, where T1FR suppresses T1F biogenesis and biofilm formation, thereby impairing bacterial adhesion and colonization. More importantly, this attenuation of bacterial virulence occurs without detectable perturbation of the gut microbiome structure. Consistently, T1FR-mediated suppression of T1F in LF82 impedes bacterial intestinal colonization and reduces the production of proinflammatory cytokines (TNFα, IL-6, and IL-8) in the intestine. Therefore, this T1FR-T1F regulatory axis provides an alternative to conventional antibiotics that does not perturb the gut microbiota, addressing both therapeutic efficacy and ecological balance in the gut.

Beyond its anti-virulence function, our comparative genomic analysis further uncovered the genetic context and evolutionary drivers underlying the global dissemination of *T1fr*. Specifically, the *T1fr* gene is predominantly encoded by antibiotic-resistant plasmids, particularly IncFII or IncFII-fusion conjugative elements. Previous studies have established that multiresistance plasmids help bacteria evade antibiotics, but this incurs fitness costs, subjecting the resistance plasmid-carrying bacterial strain to being a weak competitor in an antibiotic-free environment (32, 45, 46). Our study demonstrates that T1FR-enhanced plasmid transconjugative efficiency mitigates the adaptive costs of MDR plasmids and thus improves the growth and competitive fitness of host bacteria. This dual role drives the evolutionary adaptation of MDR plasmids by enhancing conjugation for balanced horizontal transmission and promoting vertical inheritance through fitness compensation and stable genetic maintenance. Moreover, T1FR-mediated repression of T1F may enable bacteria to detach from the intestinal epithelium and disseminate into the environment, thereby potentially accelerating the spread of resistance plasmids. Taken together, we propose that this dual-function T1FR may be crucial in driving the global dissemination of *T1fr*-positive plasmids. In addition, under antibiotic or other selective pressure, this strategy most likely prioritizes bacterial survival through enhanced plasmid transfer, facilitating the rapid acquisition of resistance traits. Plasmid-mediated fitness trade-offs are intricately linked to bacterial metabolic reprogramming, which ultimately translates into energy allocation dynamics (45, 47, 48). Okon et al. previously demonstrated that bacterial metabolism and virulence are reciprocally regulated by key metabolic transcription factors (49). Our study extends this paradigm by showing that T1FR-mediated repression of T1F and its fitness-compensatory effects are coupled to coordinated reprogramming of host metabolism, with these perturbations primarily affecting central carbon and energy metabolism.

Notably, the presence of *ltrA* in *T1fr*-positive plasmids suppressed the T1FR’s transcriptional activity. LtrA, a group II intron reverse transcriptase (RT), is a common component of virulence or resistance plasmids across *Enterobacteriaceae* (50). However, despite the widespread distribution of LtrA, its function and molecular mechanisms in plasmid biology remain uncharacterized (7, 51). In this study, we identified an intraplasmid genetic antagonism in which LtrA represses *T1fr* transcription. Furthermore, in the absence of antibiotic pressure, LtrA encoded on *T1fr*-positive plasmids abolished T1FR-mediated fitness compensation associated with resistance-plasmid carriage, while simultaneously suppressing T1FR’s ability to attenuate bacterial colonization. Strikingly, distinct antibiotic resistance gene repertoires are observed between plasmids carrying *T1fr* alone and those containing both *T1fr* and *ltrA,* indicating that the *ltrA-T1fr* axis remodels the antibiotic resistance gene map in bacteria. Collectively, this adaptation reflected an evolutionary strategy wherein genetic antagonism on mobile genetic elements fine-tunes the trade-off between colonization and resistance. This dynamic regulation prevents irreversible virulence loss and optimizes bacterial fitness in fluctuating environments.

**Fig 6:**
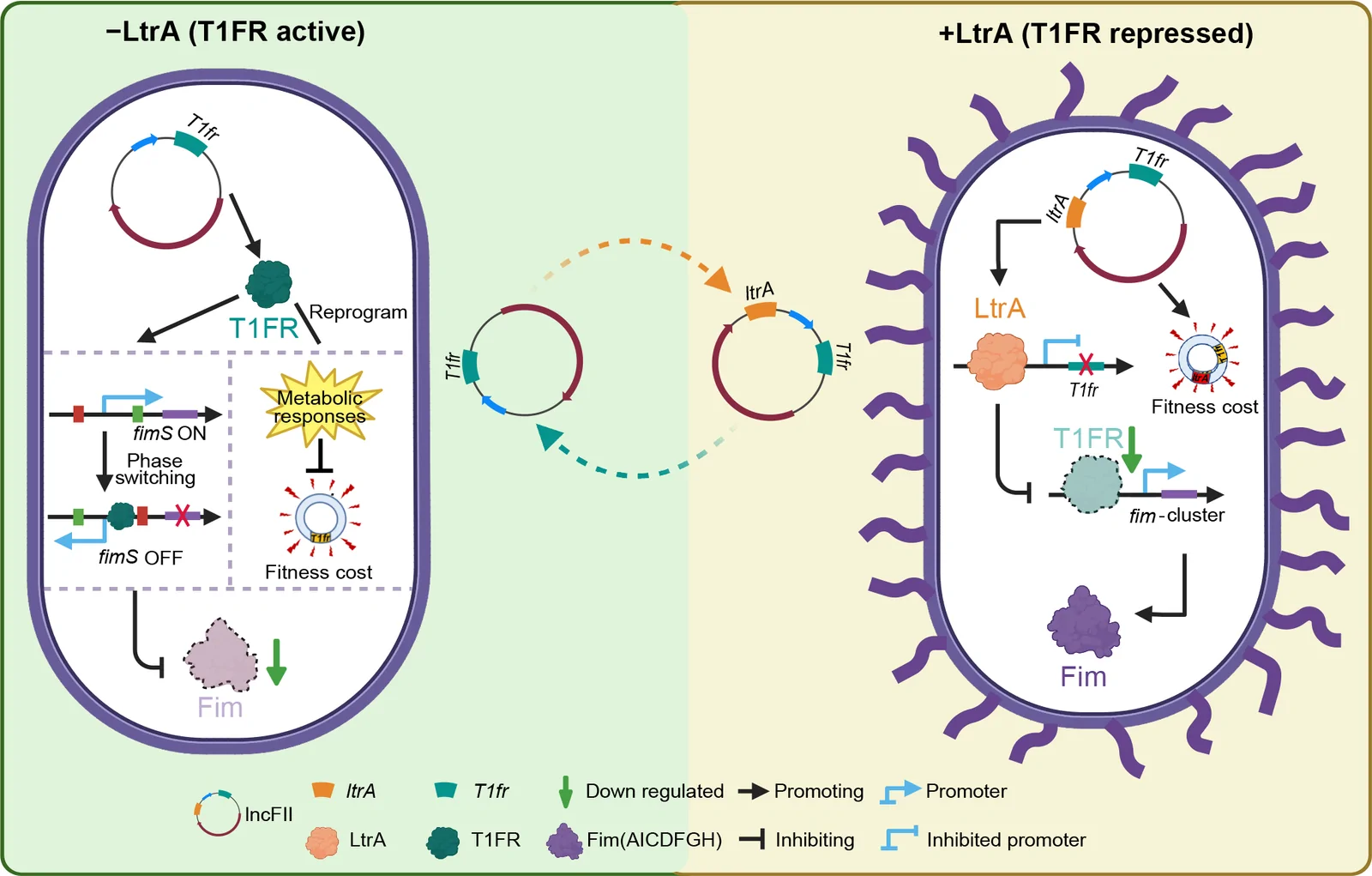
Schematic model of the T1FR system’s role in optimizing the trade-off between plasmid fitness costs and host bacterial virulence expression. T1FR, a novel identified hypothetical protein on a multiresistance IncFII plasmid, bound to the fimbrial phase variation switch (*fimS*) containing a *fimA* promoter to repress the production of T1F, causing the capacity of pathogenicity and biofilm-formation to be significantly diminished in *E. coli*. Concurrently, T1FR possesses the capacity to neutralize the fitness burden associated with vector plasmids by regulating bacterial metabolic responses. Notably, when the *ltrA* gene is co-harbored on a *T1fr*-positive plasmid, LtrA impairs T1FR function—revealing dynamic plasticity in the plasmid-mediated evolutionary trade-off between adaptive costs and pathogenic potential.

In summary, the integration of selective pressures into MGE-driven adaptive strategies underscores bacterial evolutionary resilience. The dual function of the MDR plasmid-encoded T1FR in balancing virulence attenuation (via T1F repression) and plasmid-associated fitness enhancement (via cost compensation) provides mechanistic insights into the rapid development of bacterial drug resistance under antibiotic pressure. The LtrA-T1FR regulatory axis represents a model of how MGEs orchestrate bacterial resistance, virulence, and growth to prioritize survival under complex ecological niches (Fig. 6). Crucially, identifying T1FR as a suppressor of bacterial adhesion introduces a tractable strategy to combat *Enterobacteriaceae* infections—one that circumvents the evolutionary dilemma of antibiotic resistance by disrupting bacterial colonization rather than promoting microbial killing.

## MATERIALS AND METHODS

### Bacterial strains, culture conditions, and genome sequencing

Bacterial strains and plasmids used in this study are listed in Table S3 and Table S4, respectively. All strains were cultured in lysogeny broth (LB) solid or liquid medium at 37°C. The genome sequences of the isolated strains (GCA_029087925.1) and the pEC51 plasmid (OQ230786.1) were obtained through combined splicing analysis of Illumina second-generation sequencing and Nanopore third-generation genome sequencing (52).

### Antibiotic susceptibility assays

Antibiotic susceptibility testing of the bacterial strains was investigated using the broth microdilution method, following the guidelines outlined by the Clinical and Laboratory Standards Institute (CLSI) document M02-A11 (11) and the European Committee on Antimicrobial Susceptibility Testing (EUCAST) (http://www.eucast.org, accessed on 9 May 2019). Test results were interpreted based on the CLSI document M100-S22. The *E. coli* American Type Culture Collection (ATCC) 25922 strain was utilized as the quality control strain for the above assay.

### Conjugation experiments

*E. coli* EC600 was used as the recipient bacteria to conduct conjugative transfer of plasmids by filter mating (53). LB agar plates containing rifampicin (400 µg/mL) and meropenem (2 µg/mL) were used for selection of transconjugants. The frequency of conjugative transfer was calculated as the number of transconjugants per recipient. Verification of transconjugants was performed by PCR, with primers listed in Table S5.

### Quantitative RT-PCR

Bacterial RNA was extracted using the TRIzol Max Bacterial RNA Isolation Kit (Thermo Scientific) according to the manufacturer’s instructions. RNA quality was evaluated by NanoDrop (ND-1000; Thermo Scientific) and 2% agarose gel. Reverse transcription of RNA into DNA was performed using the PrimeScript RT reagent Kit (Perfect Real Time) (Takara, Japan) according to the manufacturer’s instructions. Specific primers were designed with Primer Premier 5.0, as shown in Table S5, and RT-qPCR was performed according to previously reported methods (54). 16S rRNA was used as the normalized internal reference gene, and relative gene expression was carried out using the 2^-ΔCT^ method.

### Gene deletion to create mutants

The *ltrA* and *T1fr* gene deletions involved in this experiment were all constructed using the λRed recombination system. The native target genes were replaced by the chloramphenicol resistance gene cassette amplified from plasmid pKD3 (55). When necessary, the chloramphenicol resistance cassette was removed with plasmid pCP20. The complementation plasmids were generated by cloning the target gene into the pUC19 plasmid and verified by sequencing (56). The verified primers used for mutant creation and complementation are listed in Table S5.

### Western blot

Protein expression of the target genes on the complemented plasmid transferred into the bacterial host was confirmed by Western blotting. Bacteria were inoculated into LB broth and cultured overnight at 37°C with agitation, and the cultured bacteria were disrupted by ultrasonication. Bacterial lysates were mixed with protein loading buffer and boiled for 10 min. The samples were subjected to SDS-PAGE and then electrically transferred onto polyvinylidene difluoride membranes. The membranes were blocked with 5% skim milk for 1 h at room temperature and then incubated overnight at 4°C with Mouse anti-His-Tag mAb (Abclonal, dilution 1:5,000). The membranes were washed with PBS-Tween and incubated with horseradish peroxidase-conjugated anti-rabbit IgG antibody (Abcam, dilution 1:1,000) for 1 h at room temperature. The membranes were washed again, and proteins were detected with an imaging system (Bio-Rad ChemiDoc Touch). All antibodies are listed in Table S6.

### *fimS* phase orientation assay

The proportion of cell populations in the *fimS* switch “on/off” state was determined using a PCR-based method (31). The bacterial lysate was mixed with nuclease-free water at a ratio of 1:4 and boiled for 10 min. PCR was performed on 1 µL of the mixture, and the primers are listed in Table S5. The purified amplified product was digested with Hinf I and electrophoresed on 2% agarose gel, and the gray value of the resulting bands was determined using ImageJ. Two bands in the middle of the gel indicate that *fimS* is in the “off” orientation, while bands at the top and bottom of the gel indicate *fimS* is in the “on” orientation.

### Observation of bacterial T1F by transmission electron microscopy

Bacteria were cultured overnight at 37°C, and copper grids were used as a cell adsorption probe to be placed on a drop of bacterial suspension for 30 s. The bacterium-containing copper grids were then transferred to a droplet of 2% phosphotungstic acid and stained for 3–10 s. After washing the copper grid and draining the excess liquid with filter paper, bacterial T1F was observed using the JEM-1400FLASH transmission electron microscope (JEOL Ltd., Japan).

### *Galleria mellonella* survival assay

*G. mellonella* wax moths are routinely used as an infection model to evaluate bacterial virulence (57, 58). Bacteria were cultured to the logarithmic growth phase, and cells were harvested by centrifugation (250 × *g*, 5 min), resuspended with PBS, and adjusted to achieve an OD_600_ of 1.0 (approximately 5 × 10^8^ colony-forming units [CFUs]/mL). *G. mellonella* were randomly divided into groups of 10 larvae. The prepared bacterial solution (10 µL, 5 × 10^6^ CFUs) was injected into the larva with a micro-syringe through the left first gastropod. Two control groups were used, consisting of larvae injected with the same volume of PBS and uninjected larvae. The larvae were then cultured at a constant temperature of 37°C and protected from light for 4 days. Death of the larvae was observed and recorded every 24 h, and a survival curve was drawn using GraphPad Prism.

### Aggregation and biofilm assay

Biofilm quantification was performed by the microplate quantification assay. The bacterial solution (200 µL) with an OD600 of 0.1 was added to 96-well microplates in triplicate wells. The microplates were incubated statically at 37°C for 24 h, after which the planktonic bacteria were removed and the wells were washed with normal saline. The biofilm was fixed with ethanol (95%), followed by staining with crystal violet (0.1%) for 30 min. After removing the stain, the stained biofilm was dissolved with acetic acid (33%). Finally, the OD of the solution at a wavelength of 570 nm was quantified by the microplate reader (Thermo Multiskan go) (41).

The biofilm was also observed with a fluorescence microscope. The overnight cultured bacterial solution was diluted to 10^8^ CFU/mL, and microscope slides were immersed in the bacterial solution for 24 h. Biofilms attached to slides were washed with PBS and fixed with 2.5% glutaraldehyde. The slides were then rinsed with PBS and stained with FITC-concanavalin A (FITC-ConA) in the dark at 4°C for 30 min. After another round of washing with PBS, the slides were stained with propidium iodide in the dark for 15 min. Excess dye solution was removed by washing with PBS, followed by drying, and the biofilm was observed and analyzed with an upright fluorescence light microscope (Leica DM4 B).

### Adhesion of bacteria to eukaryotic cells

The adhesion capacity of the strains was analyzed using RAW264.7 and HIEC-6 cell lines. RAW264.7 (ATCC TIB-71) and HIEC-6 (ATCC CRL-3266) cells were grown in Dulbecco’s modified Eagle’s medium (Gibco) and RPMI 1640 medium (Sigma-Aldrich), respectively. Both cell lines were incubated at 37°C in a humidified 5% CO2 atmosphere for the indicated durations (12 h for RAW264.7; 24 h for HIEC-6). The cultivated cells were digested with trypsin and distributed into 24-well plates, with 4 × 10^5^ cells per well, and cultured for an additional 16 h. Non-adherent cells were then washed away using PBS. The bacteria cultured overnight were collected by centrifugation at 10,000 × *g* in centrifuge tubes and resuspended with medium. The resuspension was dispensed into the 24-well plates containing the eukaryotic cells, at a multiplicity of infection ratio of 10 and incubated at 37°C for 3 h (59). The liquid in the wells was discarded, and the free cells were washed with PBS. Bacterial adsorption capacity was evaluated using an upright fluorescence microscope, while the cells were resuspended in PBS at 10-fold serial dilutions and spread onto LB agar plates to determine bacterial counts.

### Protein purification

*E. coli* BL21(DE3) carrying pET28a-*ltrA*-his or pET28a-*T1fr*-his were cultured at 37°C until an OD_600_ of about 0.6 was achieved. At this point, the cells were induced overnight at 30°C with 1 mM isopropyl-β-D-thiogalactoside. The bacteria, collected by centrifugation, were resuspended in lysis buffer (8 M urea, 100 mM NaH_2_PO_4_, and 10 mM Tris-HCl, at pH 8.0), and the cells were lysed by sonication. The lysed suspension was centrifuged (4°C, 12,000 × *g*) to collect the supernatant. Proteins were purified by Ni-NTA Resin (Thermo Fisher Scientific, USA) affinity chromatography according to the manufacturer’s instructions. After extensive washing with buffer containing 60 mM imidazole, proteins were eluted with 500 mM imidazole eluent. The proteins were assessed using 15% SDS-PAGE, and concentrations were measured with the Bradford protein assay kit (Takara, Japan) according to the instructions.

### Electrophoretic mobility shift assay

The binding of LtrA (the group II intronic reverse transcriptase) to the *T1fr* promoter and T1FR to *fimS* containing the *fimA* promoter were assessed by EMSA. The *T1fr* promoter was predicted using BPROM (22, 23). The DNA fragment corresponding to the promoter sequence fused to a biotin tag was synthesized by Sangon Biotech (Shanghai, China), and the reference sequence was a fragment missing the predicted binding region (24, 25, 27). The oligonucleotide sequences are listed in Table S7 and Table S8. The binding between protein and DNA was evaluated with the EMSA kit (Thermo Fisher Scientific, USA) according to the instructions. DNA, at a final concentration of 100 nM, and equal increasing amounts of protein were added to binding reaction buffer (50 mM Tris-HCl [pH 7.5], 100 mM NaCl, 0.4 mM EDTA, and 0.1% NP-40) and incubated at room temperature for 30 min. The incubated mixture was subjected to 6% native polyacrylamide gel electrophoresis (80 V, 30 min) in 53Hi-Density TBE buffer, followed by membrane transfer. Detection was performed using a chemiluminescent nucleic acid detection module (Thermo Fisher Scientific, USA) according to the instructions.

### Oligo pull-down

Streptavidin-conjugated magnetic beads (NEB) were pre-washed twice using binding buffer (50 mM Tris-HCl pH 7.5, 150 mM NaCl, 0.4 mM EDTA, 1% NP-40, and 1 mM DTT). LtrA or T1FR protein was pre-cleared with 1/10 pre-washed streptavidin-conjugated magnetic beads. Meanwhile, the remaining streptavidin-conjugated magnetic beads were also pre-cleared by incubation with binding buffer containing 0.2 mg/mL bovine serum albumin (Amresco) for 1 h at 4°C under gentle rotation. Then, the LtrA or T1FR protein and the streptavidin-conjugated magnetic beads were incubated together with biotin-labeled DNA oligonucleotides at 4°C for 2 h under gentle rotation. Subsequently, the bead-oligo-protein mixture was washed five times with binding buffer and heated in NuPAGE LDS Sample Buffer (4×) (Invitrogen) at 95°C for 10 min. Finally, the binding ratio was calculated as the intensity of the pulled-down T1FR protein band divided by the intensity of the total input protein band.

### Mouse infection model and histopathological examination

The animal experimental procedures conducted in this study were carried out in accordance with the guidelines set by the Animal Ethics Committee (AEC) of the College of Life Sciences, Sichuan University (license no. K2022018). BALB/C mice were purchased from Chengdu Dashuo Experimental Animal Co., Ltd. All animals were maintained under standardized temperature- and light-controlled conditions (12-h light/dark cycle) in individually ventilated cages with companion mice with free access to food and water.

Mice aged 5 weeks were randomly divided into 4 groups, with 10 mice in each group, and infected with 10^9^ CFU of strain LF82 with or without T1FR or appropriate amounts of PBS for 3 consecutive days by intragastric administration (60, 61). On day 7, the intestines of mice in different groups were excised and examined by tissue imaging, while colon tissues were collected and fixed with 4% paraformaldehyde for H&E or Alcian blue staining. The stained sections were imaged by the panoramic tissue section scanning system (Olympus VS200) for pathological diagnosis. The intestinal contents of the mice were collected, and the microbial compositions were evaluated by Sangon Biotech (Sangon Biotech, Shanghai, China) using 16S rRNA sequencing. The content-cleared colorectal tissue was homogenized in PBS (0.1 g/mL). The homogenate was then serially diluted 10-fold and applied to LB agar plates containing tetracycline (5 μg/mL) to assess the bacteria’s colonization capacity. The T1F of the AIEC strain induced increased secretion of cytokines in human cells (41). Therefore, homogenates of colon tissues were collected to determine cytokine (TNFα, IL-6, and IL-8) content, following the instructions of the mouse-specific ELISA kit (Jiangsu Meimian Industrial Co., Ltd, China).

### Bioinformatics analysis of *T1fr* carriage and genetic context

The whole-genome sequences of strains carrying the *ltrA* or *T1fr* gene were downloaded from the BioSample database and aligned, and the construction and visualization of bacterial evolutionary trees were performed by CGE CSI Phylogeny 1.4 and iTOL v6, respectively (62). Sequence types of the strains were analyzed by using Multi Locus Sequence Typing v2.23.0 (63). The genome sequences of plasmids carrying the *ltrA* or *T1fr* gene were downloaded from the GenBank database and aligned. Plasmid replicon types were confirmed using PlasmidFinder 2.1 (64). The plasmid nucleic acid sequence included the *T1fr* gene and 9,000 bp of its upstream and downstream sequences, and an evolutionary tree was constructed. Eight sequences with different evolutionary distances were selected and compared with the genetic background of the *T1fr* gene using EasyFig 2.1 (65). Antibiotic genes in plasmids were identified using ResFinder (66).

### Growth curves

The strains were inoculated into LB liquid medium and incubated overnight at 37°C with shaking at 180 rpm. The bacterial solution was diluted with LB liquid medium until the OD_600_ reached 0.004. Subsequently, the diluted bacterial solution was inoculated into LB liquid medium at a ratio of 1:1,000 and incubated with shaking at 37°C for 13 h. Growth curves were constructed based on the hourly measurement of OD_600_ values obtained for the bacterial solution.

### Bacterial competition

The bacterial culture grown overnight was diluted until the OD_600_ value reached 0.004. The diluted bacterial culture of competitive paired strains (CFIIΔ*ltrA* vs CFII, CFIIΔ*ltrA*Δ*T1fr* vs CFIIΔ*ltrA*) was mixed at a 1:1 ratio. The mixed bacterial solution was inoculated into LB medium (1:1,000), cultured with shaking at 37°C, and bacteria were allowed to compete and grow for 8 days (67). On each day throughout the experiment, 10-fold serial dilutions of cultured bacteria were spread onto LB agar plates containing rifampicin (400 µg/mL), meropenem (2 µg/mL), or chloramphenicol (16 µg/mL) to screen for corresponding resistant strains. The formula *S* = ln (number of colonies of one strain/number of competitors) was applied to evaluate the competitive advantages of the two strains. A fitness advantage was judged if *S* > 0 (57).

### RNA-Seq transcriptome analysis

Bacterial cells in the logarithmic phase were collected, and total bacterial RNA was extracted using the TRIzol Max Bacterial RNA Isolation Kit (Thermo Scientific) according to the manufacturer’s instructions. Quality control analysis and RNA sequencing were performed by Majorbio (Majorbio Bio-pharm Technology Co., Ltd., Shanghai, China) and Sangon (Sangon Biotech, Shanghai, China). The *E. coli* sequences GCA_000005845.2 and GCA_016803855.1 downloaded from GenBank were utilized as the reference genomes. Analysis of differentially expressed genes was conducted using the DESeq2 package.

### Untargeted metabolomics analysis

The bacterial cells cultured to the logarithmic growth phase were quickly frozen in liquid nitrogen and stored at −80°C for metabolite detection. Metabolomic assays and integrative analysis of the transcriptome and metabolome were conducted by MetWare (MetWare Biological Science and Technology Co. Ltd., Wuhan, China). The metabolites were detected using the UPLC-ESI-MS/MS system. The self-compiled database MWDB (MetWare Biological Science and Technology Co. Ltd., Wuhan, China) was used as a search library for qualitative analysis of primary and secondary MS data (68). Differentially accumulated metabolites were filtered out based on the variable importance of the projection ≥1 and | log_2_(fold change) | ≥1 (68). The integrative analysis of the metabolome and transcriptome was based on gene expression. The Gene Ontology and Kyoto Encyclopedia of Genes and Genomes functional pathway enrichment analysis of differential genes and metabolites was performed using David (https://davidbioinformatics.nih.gov/).

### Statistical analyses

Statistics underwent one-way analysis of variance (ANOVA). Homoscedasticity was confirmed through Levene’s test prior to ANOVA, and differences among the means were assessed by Duncan’s multiple-range test using SPSS Statistics 22.0 (SPSS Inc. USA). The results were presented as mean ± standard error of the mean (S.E.M). Differences in data between groups were considered significant when *P* < 0.05. Data graphs were drawn using GraphPad Prism 8.3 (GraphPad Software Inc., USA), “*” indicates *P* < 0.05, “**” indicates *P* < 0.01, and “***” indicates *P* < 0.001.

## Data Availability

RNA-seq data generated in this study have been deposited in the Genome Sequence Archive of National Genomics Data Center under accession code CRA014579 (linked to the BioProject with accession no. PRJCA023018). For microbiota, the raw data sets are available in the EBI database system under project accession number PRJNA909031. All other data supporting the findings reported herein are available on reasonable request from the corresponding author.

## SUPPLEMENTAL MATERIAL

Supplemental figures mbio.01899-26-s0001.pdf Fig. S1-S10.

Supplemental tables mbio.01899-26-s0002.pdf Tables S1-S8.

ASM does not own the copyrights to Supplemental Material that may be linked to, or accessed through, an article. The authors have granted ASM a non-exclusive, world-wide license to publish the Supplemental Material files. Please contact the corresponding author directly for reuse.
