# Supplementary material for: A plasmid-encoded genetic switch orchestrates the bacterial virulence-fitness trade-off to drive antibiotic resistance dissemination
Source: mBio. 2026 Sep 16;17(10):e01899-26. doi: 10.1128/mbio.01899-26 (PMC13712751; doi:10.1128/mbio.01899-26)
Supplement: Supplementary file 1 [file mbio.01899-26-s0001.pdf]

Supplemental Figures (S1-S10)

A plasmid-encoded genetic switch balances virulence and fitness costs to drive global dissemination of antibiotic resistance

Hongcheng Wei [a, b, c, e #], Wenlan Yang [d #], Tiejun Zhang [a, b #], Wei Xu [a, b #], Ke Wu [a, b], Linghan Kong [a, b], Hao Li [a, b], Kailu Wang [a, b], Wenjun Yan [a, b], Ming Yang [a, b], Changyu Zhou [a, b], Songhua Li [d], Zhonghan Li [a], Changwei Lei [a, b *], Hongning Wang [a, b *]

[a] Laboratory of Bio-Resource and Eco-Environment of Ministry of Education, College of Life Sciences, Sichuan University, Chengdu, 610064, Sichuan Province, China

[b] Animal Disease Prevention and Food Safety Key Laboratory of Sichuan Province, Chengdu, 610064, China

[c] School of Laboratory Medicine, Chengdu Medical College, Chengdu, 610500, Sichuan Province, China

[d] Inner Mongolia Key Laboratory for Molecular Regulation of the Cell, College of Life Sciences, Inner Mongolia University, Hohhot 010021, China

[e] Chengdu Academy of Educational Sciences, 610036, Sichuan Province, China

[#] These authors contributed equally to this work

[*] Corresponding E-mail address: leichangwei@scu.edu.cn; hongningwang@scu.edu.cn.

Corresponding physical mailing address: 24 South Section 1, First Ring Road, Chengdu City, Sichuan Province, China This Supplementary data information contains:

- Supplementary Figures (**S1-S10)**

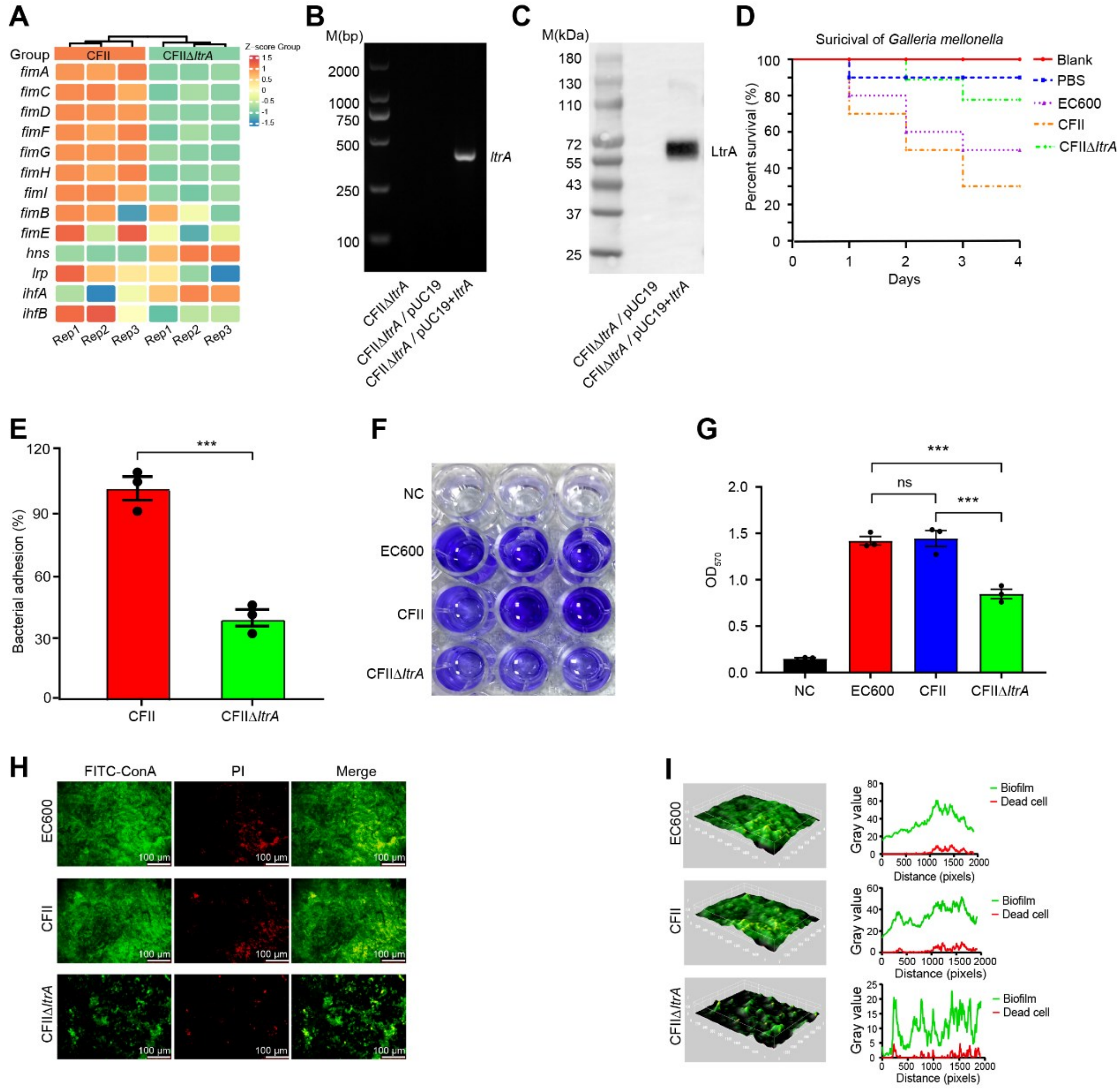

Fig. S1 The pEC51 plasmid-encoded *ltrA* gene maintains virulence and biofilm formation in host bacteria.

(A) Quantitative comparison of fimbriae structural genes and regulatory genes expression *via* RT-qPCR analysis in CFIIΔ*ltrA* versus CFII. $n = 3$.

(B) The expression of the exogenous *ltrA* gene in CFIIΔ*ltrA* strain was detected by PCR and gel electrophoresis. Both strains of CFII and CFIIΔ*ltrA* / pUC19 as control group.

(C) Surface fimbriae characteristics of CFIIΔ*ltrA*/pUC19 (vector control) and CFIIΔ*ltrA*/pUC19+*ltrA* using electron microscopy. Scale bar, 2 μm or 500 nm.

(D) Identification of the virulence and lethality of three bacterial strains EC600, CFII and CFIIΔ*ltrA* using the *G. mellonella* infection models. Both blank and PBS as control groups.

(E) HIEC-6 adhesion assays of cell adhesion ability of CFIIΔ*ltrA* compared to CFII. Wild-type

EC600 (without plasmid) was included as the baseline control, “*” indicates $P < 0.05$, “**” indicates $P < 0.01$, “***” indicates $P < 0.001$, two-tailed student’s t test. Data represent mean ± SEM of three independent experiments.

(F) The biofilm formation capacities of three bacterial strains EC600, CFII and CFIIΔ*ltrA* were detected by observing of differences in formation of crystal violet-stained biofilms.

(G) Quantitative spectrophotometric analysis of crystal violet-stained biofilms in Fig. S1F. “*” indicates $P < 0.05$, “**” indicates $P < 0.01$, “***” indicates $P < 0.001$, two-tailed student’s t test. Data represent mean ± SEM of three independent experiments.

(H) The biofilm formation capacities of three bacterial strains EC600, CFII and CFIIΔ*ltrA* were detected by fluorescence microscopy. Scale bar, 100 μm.

(I) Three-dimensional reconstruction and quantitative analysis of biofilms formed by EC600, CFII and CFIIΔ*ltrA* strains.

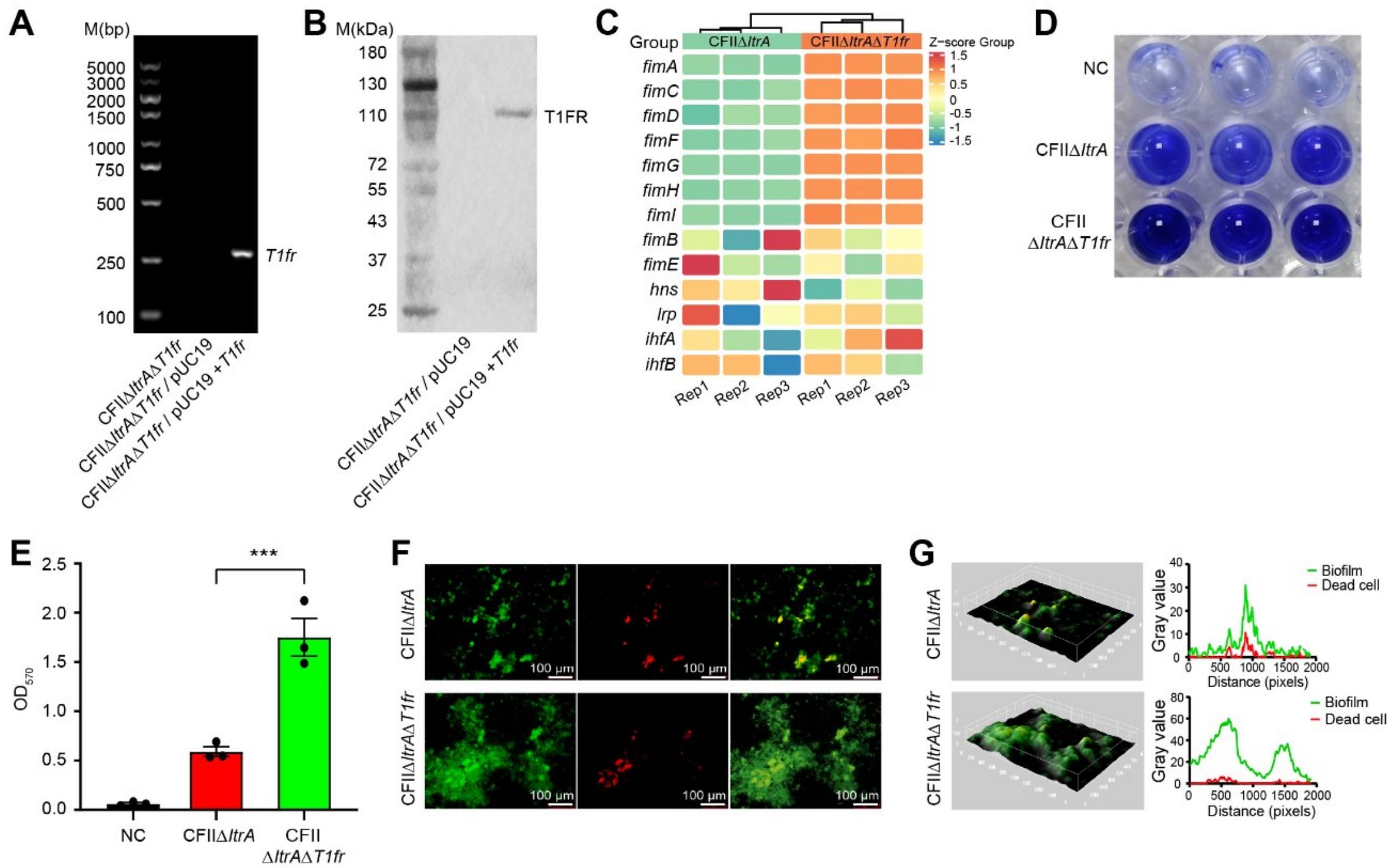

Fig. S2 *T1fr* deficiency in CFIIΔ*ltrA* promotes *fim*-cluster genes expression and biofilm formation in *E. coli*.

(A) The expression of the exogenous *T1fr* gene in CFIIΔ*ltrA*Δ*T1fr* strain was detected by PCR and gel electrophoresis. Both strains of CFIIΔ*ltrA*Δ*T1fr* and CFIIΔ*ltrA*Δ*T1fr*/ pUC19 as control group.

(B) The expression of the exogenous *T1fr* gene in CFIIΔ*ltrA*Δ*T1fr* strain was confirmed by western blot.

(C) Quantitative comparison of fimbriae structural genes and regulatory genes expression *via* RT-qPCR analysis in CFIIΔ*ltrA*Δ*T1fr* strain versus CFIIΔ*ltrA* strain. $n = 3$.

(D) Biofilm formation capacities of both strains CFIIΔ*ltrA* and CFIIΔ*ltrA*Δ*T1fr* were detected by observing of differences in formation of crystal violet-stained biofilms.

(E) Quantitative spectrophotometric analysis of crystal violet-stained biofilms (D). "*" indicates $P < 0.05$, "**" indicates $P < 0.01$, "***" indicates $P < 0.001$, two-tailed student's t test. Data represent mean ± SEM of three independent experiments.

(F) The biofilm formation capacity of CFIIΔ*ltrA* and CFIIΔ*ltrA*Δ*T1fr* assessed by fluorescence microscopy. Scale bar, 100 μm.

(G) Three-dimensional reconstruction and quantitative analysis of biofilms formed by CFIIΔ*ltrA* and CFIIΔ*ltrA*Δ*T1fr* strains.

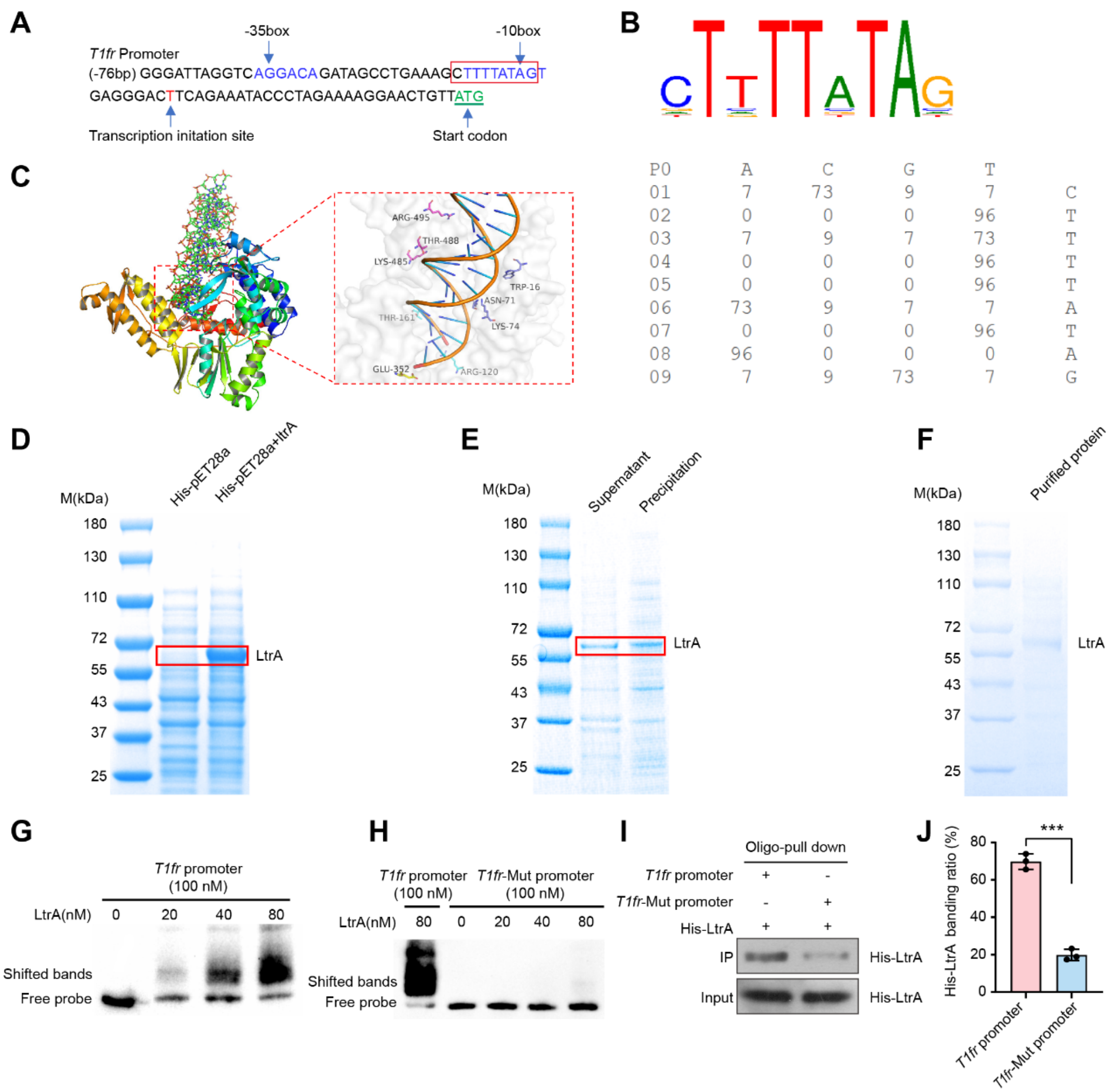

Fig. S3 LtrA binds to the *T1fr* promoter.

(A) Prediction of the binding motif of LtrA protein on the promoter of *T1fr*.

(B) Molecular docking simulation of LtrA-*T1fr* interactions. Predicted binding conformation showing LtrA (surface representation) bound to *T1fr* promoter (orange cartoon).

(C) The LtrA protein expression was detected by SDS-PAGE gel electrophoresis.

(D) Distribution of LtrA protein in the supernatant or cell preparation of BL21(DE3) strain.

(E) The LtrA purified protein expression was detected by SDS-PAGE gel electrophoresis.

(F) Sequence analysis diagram showing the prediction of the T1FR promoter. Blue letters indicated -35box and -10box; Red letter indicated transcription initiation site; Green letters indicated start codon; The red box indicated the putative binding region of LtrA protein.

(G) The binding efficiency of LtrA purified proteins with *T1fr* promoter were detected by

electrophoretic mobility shift assay (EMSA).

(H) The binding efficiency of LtrA purified proteins with *T1fr*-Mut promoter (deletion putative LtrA binding region) were detected by EMSA.

(I) LtrA protein was pulldown by *TIfr* promoter. The expression of each group was confirmed by Western blotting using the anti-His antibody.

(J) Quantification of the gray signals of the bands in the western blot of Fig. S3I. "*" indicates $P < 0.05$, "**" indicates $P < 0.01$, "***" indicates $P < 0.001$, two-tailed student's *t* test. Data represent mean ± SEM of three independent experiments.

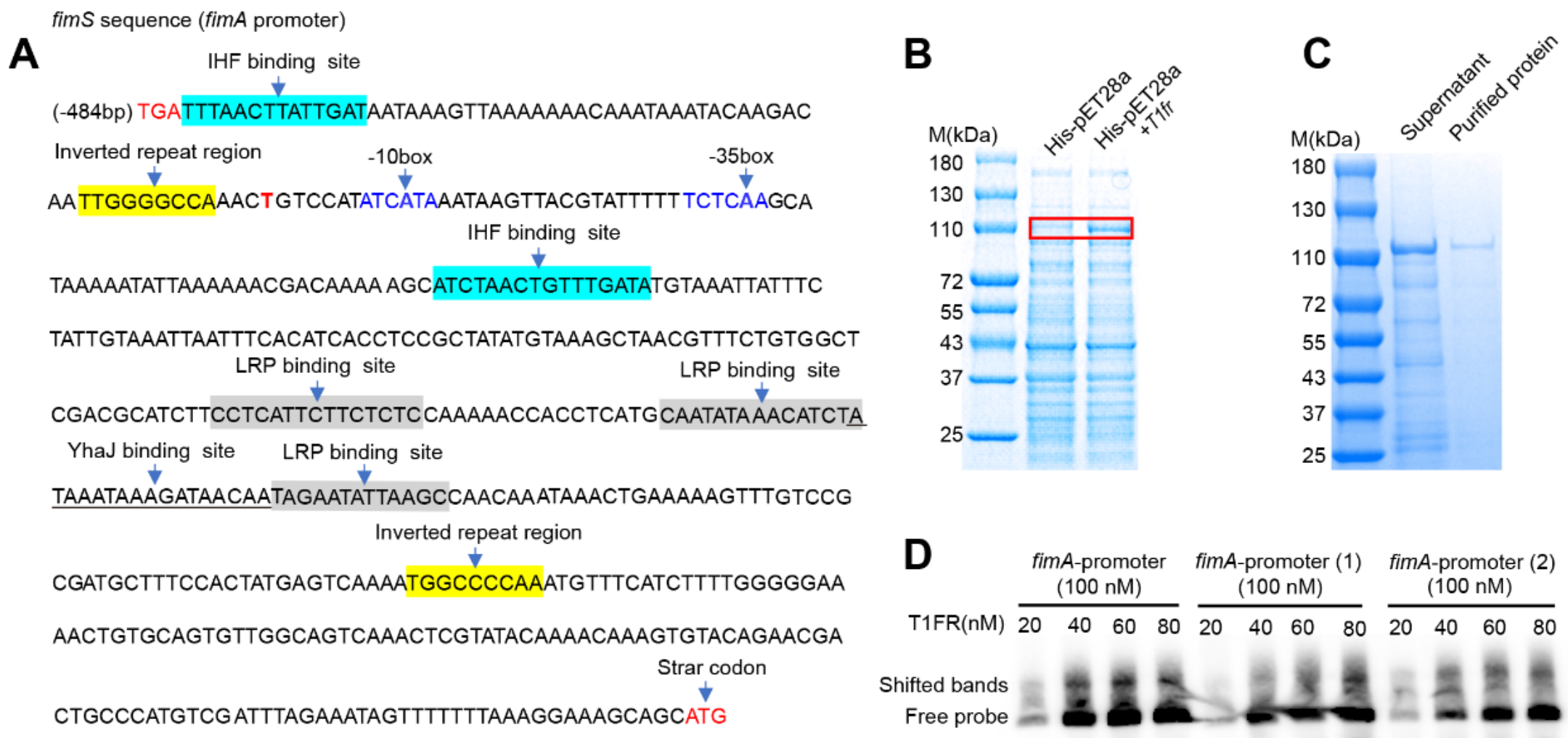

Fig. S4 Prediction of the binding of T1FR protein to the *fimS*.

(A) The sequence of TIF phase variation converter *fimS* in *Escherichia coli*. Gray shading indicated LRP binding site; Yellow shading indicated inverted repeat region; Sky blue shading indicated IHF binding site; The underlined region represented YhaJ binding site; the red box indicated the putative binding region of T1FR protein; and green letters indicated start codon.

(B) The T1FR protein expression was detected by SDS-PAGE gel electrophoresis.

(C) Distribution of T1FR protein in the supernatant or cell preparation of BL21(DE3) strain.

(D) The binding efficiency of *fimS* truncated sequence and T1FR purified protein was detected by electrophoretic mobility shift assay (EMSA).

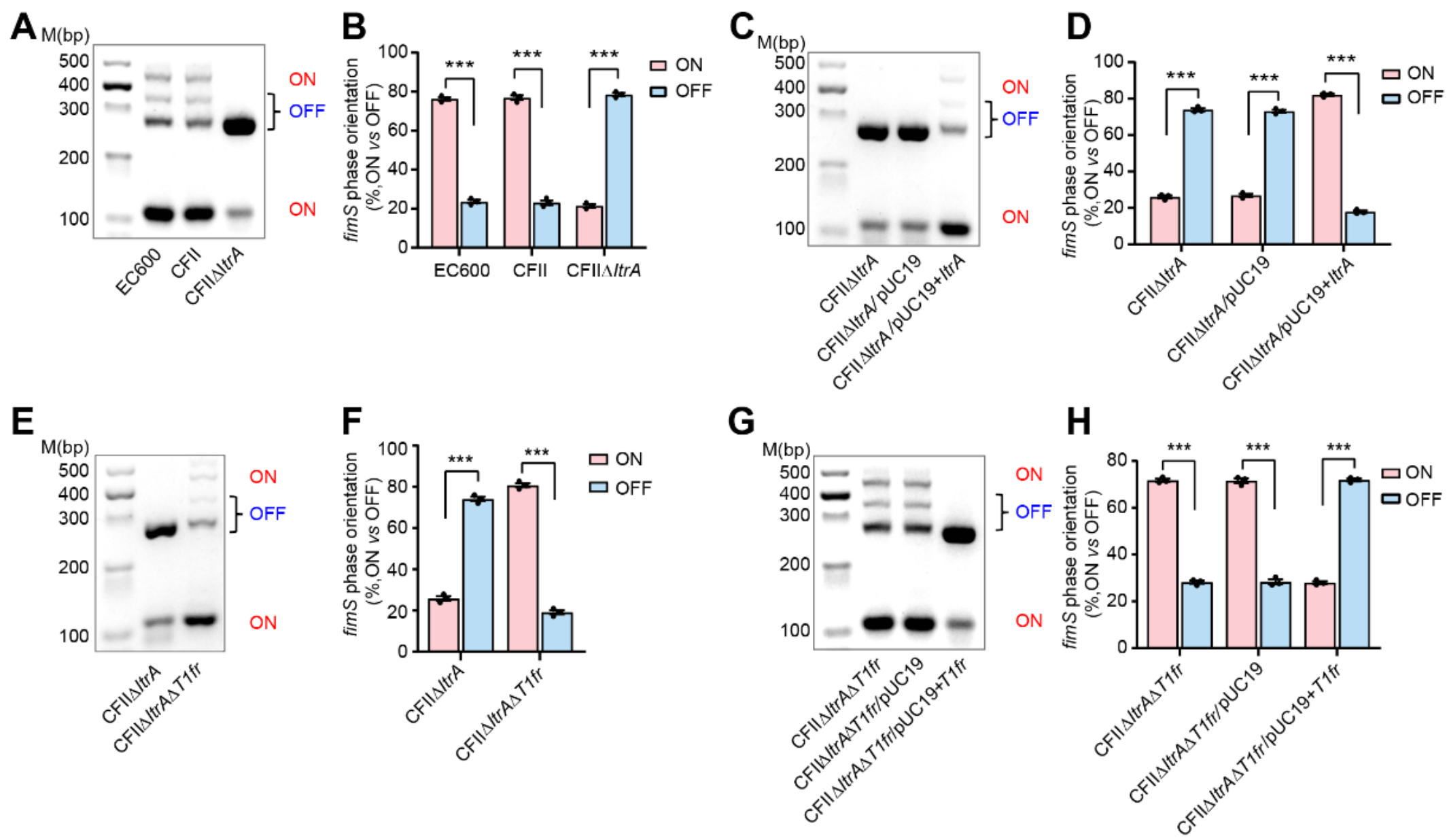

Fig. S5 Reorientation transition of *fimS* in host bacteria carrying plasmid pEC51.

(A) Detection of *fimS* orientation in EC600 strains harboring pEC51 plasmid or *ltrA*-negative pEC51 plasmid. Top/bottom bands indicate the "ON" orientation; middle bands indicate the "OFF" orientation.

(B) The quantitative analysis corresponding to Figure S5A.

(C) Detection of *fimS* orientation in EC600 strain with or without harboring *ltrA*-containing pUC19 plasmid. Top/bottom bands indicate the "ON" orientation; middle bands indicate the "OFF" orientation.

(D) The quantitative analysis corresponding to Figure S5C.

(E) Detection of *fimS* orientation in EC600 strains harboring with or without *T1fr* gene on *ltrA*-negative plasmid pEC51. Top/bottom bands indicate the "ON" orientation; middle bands indicate the "OFF" orientation.

(F) The quantitative analysis corresponding to Figure S5E.

(G) Detection of *fimS* orientation in EC600 strains harboring *ltrA* and *T1fr* double negative pEC51 plasmid with *T1fr* complementation. Top/bottom bands indicate the "ON" orientation; middle bands indicate the "OFF" orientation.

(H) The quantitative analysis corresponding to Figure S5G.

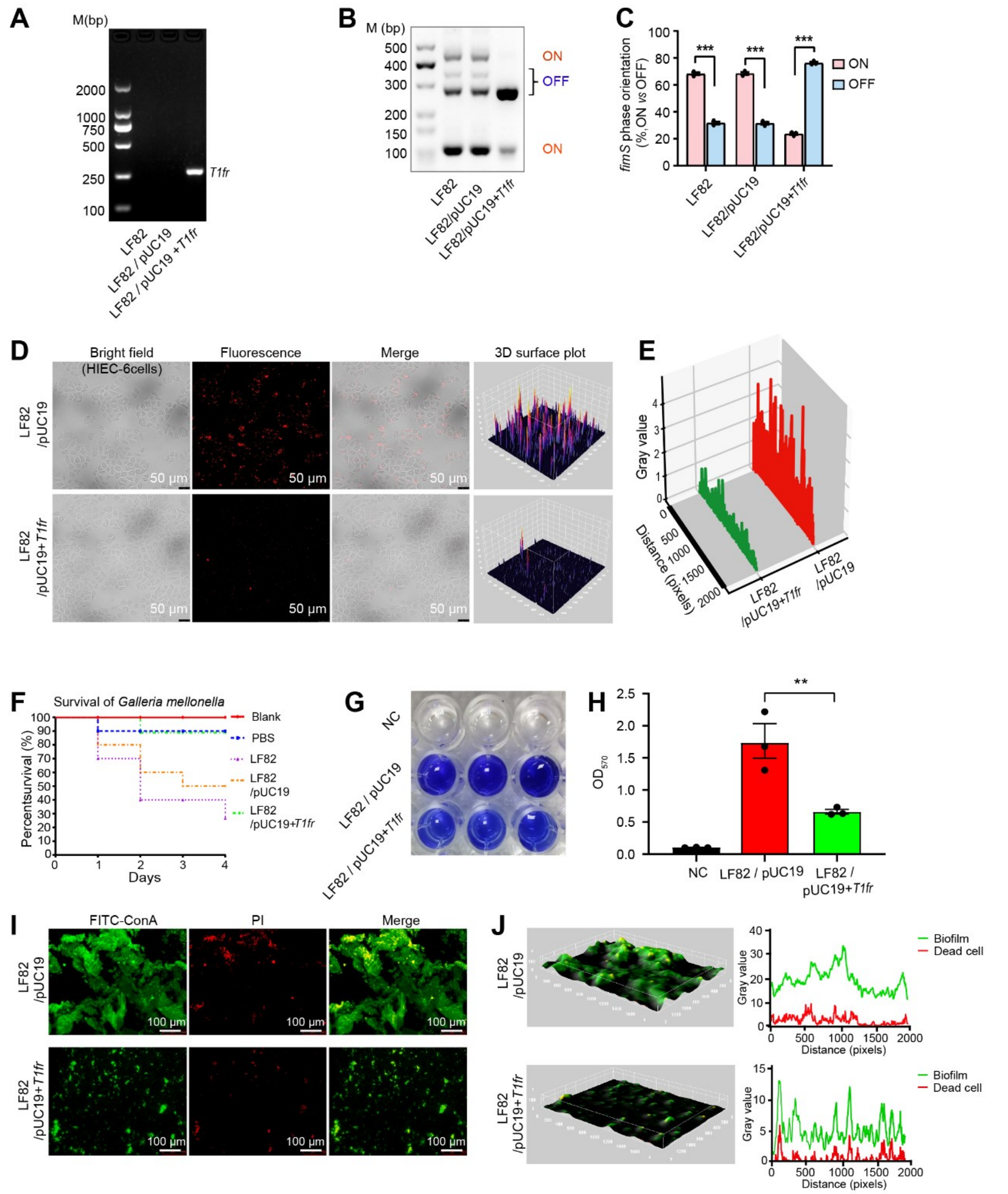

Fig. S6 T1FR reduces adhesion of LF82 to eukaryotic cells.

(A) The presence of the exogenous *T1fr* gene in LF82 was confirmed by PCR and gel electrophoresis.

(B) *fimS* orientation was analyzed in LF82, LF82/pUC19, or LF82/pUC19+*T1fr* strains.

(C) Proportional quantification of *fimS* orientation in LF82, LF82/pUC19, or LF82/pUC19+*T1fr*.

(D) Adhesion capacity of LF82/pUC19+*T1fr* and LF82/pUC19 (vector control) strains onto HIEC-

6 cells was detected by cell fluorescence assays. The bacteria were labeled with mCherry. Scale bar, 50 μm.

(E) Quantitative analysis of adhesion ability for LF82/pUC19+*T1fr* or LF82/pUC19 strains onto HIEC-6 cells using ImageJ (Fuji) software to characterize the signal of mCherry in Fig. S6D. The LF82/pUC19+*T1fr* and LF82/pUC 19 strains both expressed mCherry signaling.

(F) The lethality of the host bacteria infected with LF82, LF82/pUC19 and LF82/pUC19+*T1fr* strains were analyzed by *G. mellonella* survival assays. PBS group functioned as the blank control.

(G) Biofilm formation capacities of both strains LF82/pUC19 and LF82/pUC19+*T1fr* were detected by observing of differences in formation of crystal violet-stained biofilms.

(H) Quantitative spectrophotometric analysis of crystal violet-stained biofilms (Fig. S6G) revealed the differences in biofilm formation of LF82/pUC19+*T1fr* and LF82/pUC19. "*" indicates $P < 0.05$, "**" indicates $P < 0.01$, "***" indicates $P < 0.001$, two-tailed student's *t* test. Data represent mean ± SEM of three independent experiments.

(I) Biofilm formation capacity of LF82/pUC19 and LF82/pUC19+*T1fr* strains was assessed by fluorescence microscopy. Scale bar, 100 μm.

(J) Three-dimensional reconstruction and quantitative analysis of biofilms formed by LF82/pUC19 and LF82/pUC19+*T1fr* strains.

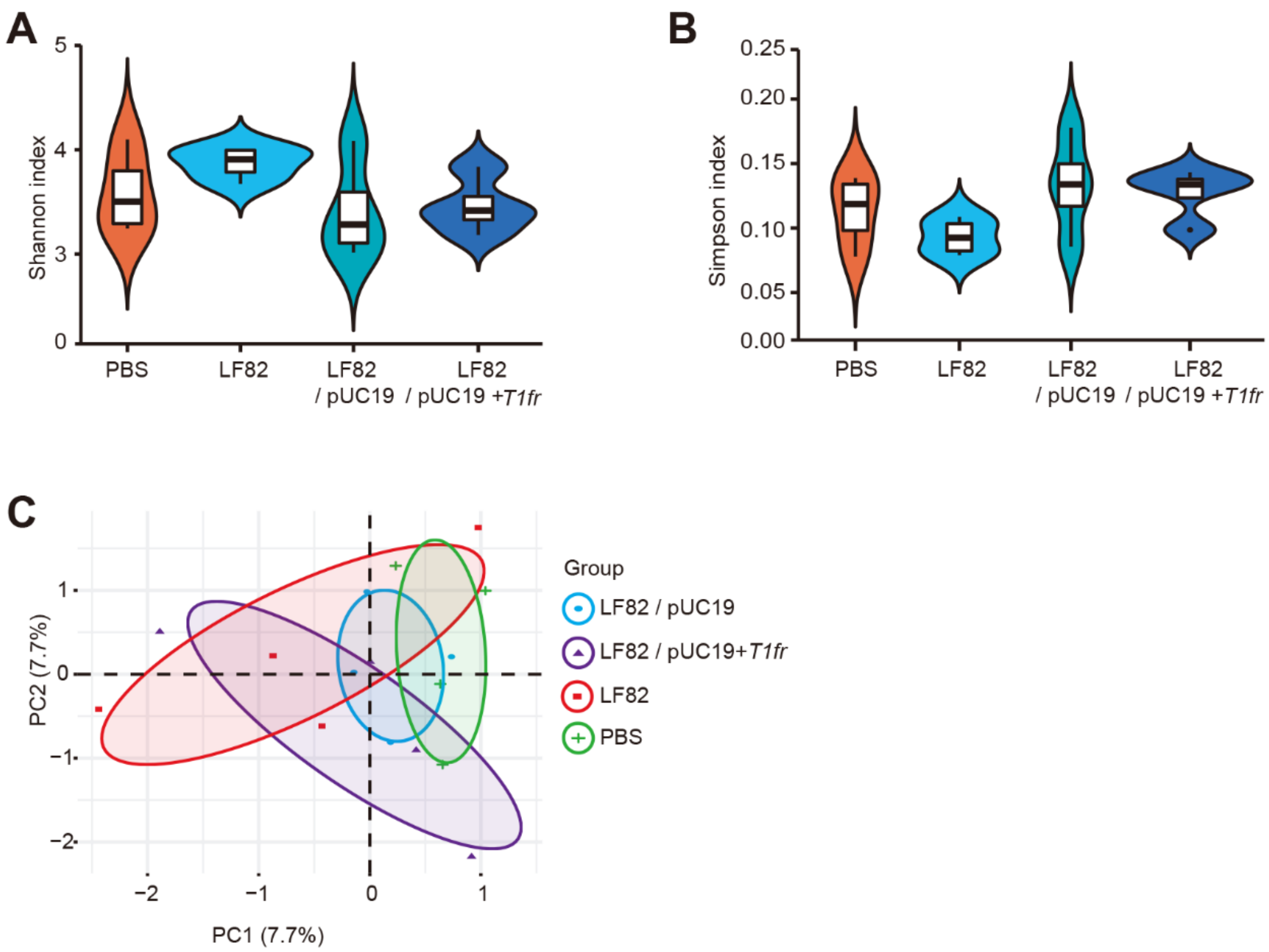

Fig. S7 T1FR expression does not affect intestinal microbiota composition.

(A) Violin chart showing the Shannon index of intestinal microbiota in murine models infected by LF82/pUC19+*T1fr* and LF82/pUC19. Fecal samples were collected at day 7 post-infection (*n* = 5 mice per group).

(B) Violin chart showing the Simpson index of intestinal microbiota in murine models infected by LF82/pUC19+*T1fr* and LF82/pUC19 (vector control). Fecal samples were collected at day 7 post-infection (*n* = 4 per group).

(C) *β*-diversity analysis of intestinal microbiota. Principal Component Analysis plot based on [distance metric] showing microbial community differences in mice infected with LF82, LF82/pUC19, or LF82/pUC19+*T1fr* strains, PBS as blank control. *n* = 4 mice per/group.

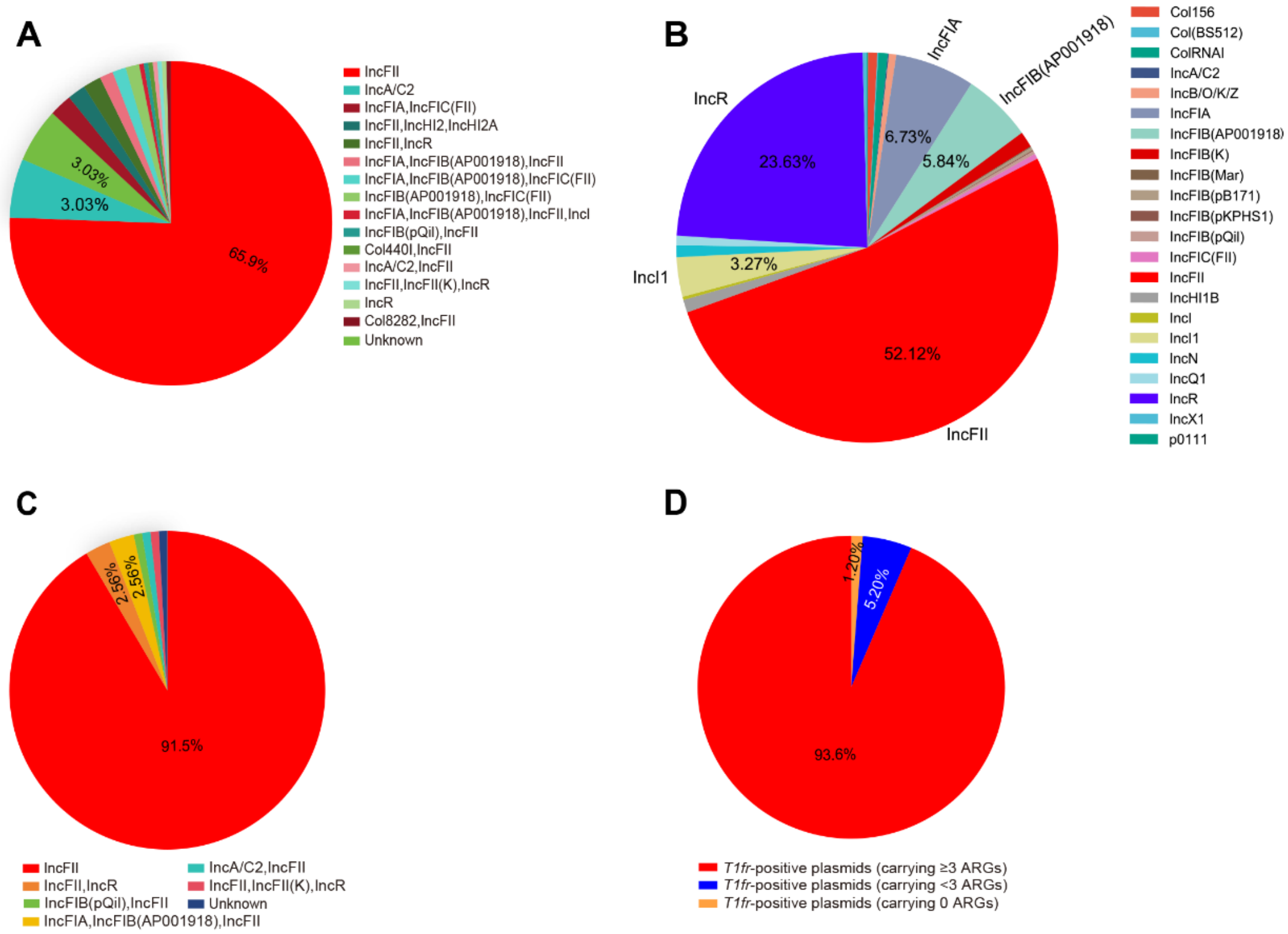

Fig. S8 The genetic context and carrying vector of T1FR

(A) Types and proportions of *T1fr*-positive plasmids obtained from the GenBank database or archived in our laboratory.

(B) Types and proportions of *ltrA*-positive plasmids obtained from the GenBank database or archived in our laboratory.

(C) Pie diagram showing the types and proportions of plasmids harboring *ltrA* and *T1fr* genes (plasmids downloaded from the BioSample database or archived in our laboratory).

(D) Proportion of *T1fr*-positive plasmids harboring varying numbers of resistance genes.

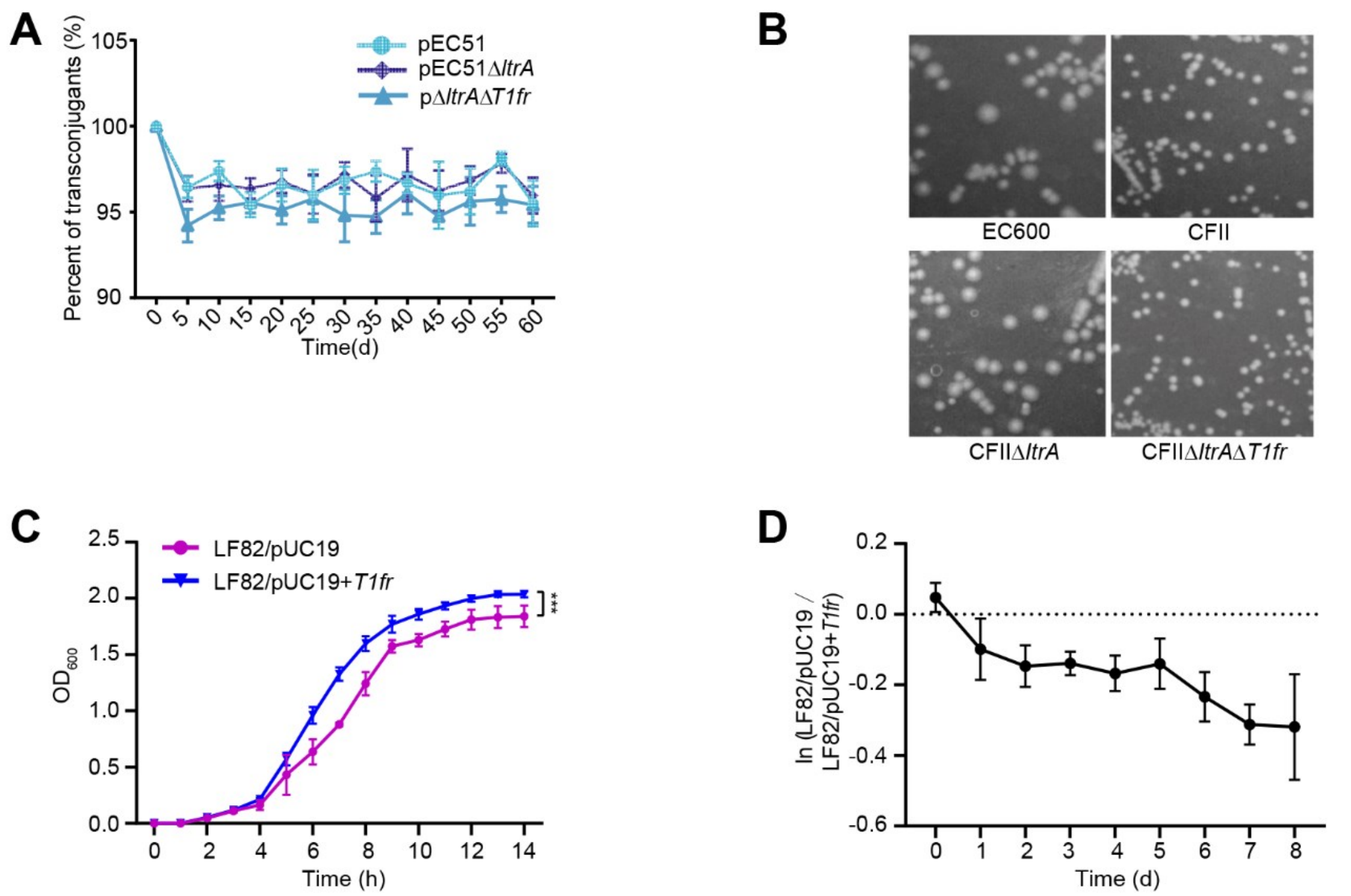

Fig. S9 Growth improvement in *E. coli* mediated by *T1fr* gene

(A) Plasmid stability assays over approximately 600 of continuous cultivation. Long-term evolutionary stability of wild-type IncFII plasmids (pEC51), *ltrA*-deficient mutants (pEC51Δ*ltrA*), and *ltrA*/*T1fr* double-deletion variants (pEC51Δ*ltrA*Δ*T1fr*) was monitored in bacterial populations ($n = 3$).

(B) Clonal morphology of *E. coli* with or without normal *T1fr* gene expression. The different treated group strains grown on plates for 12 hours.

(C) The growth curves of LF82 strains with or without T1fr gene expression. $n = 3$. *** indicates $P < 0.001$, two-tailed student's *t* test.

(D) Competitive fitness of LF82 strains with or without the *T1fr* gene. Population dynamics of LF82/pUC19 strains versus LF82/pUC19+*T1fr* strains during 8 days of co-culture (mean ± SEM, $n = 3$).

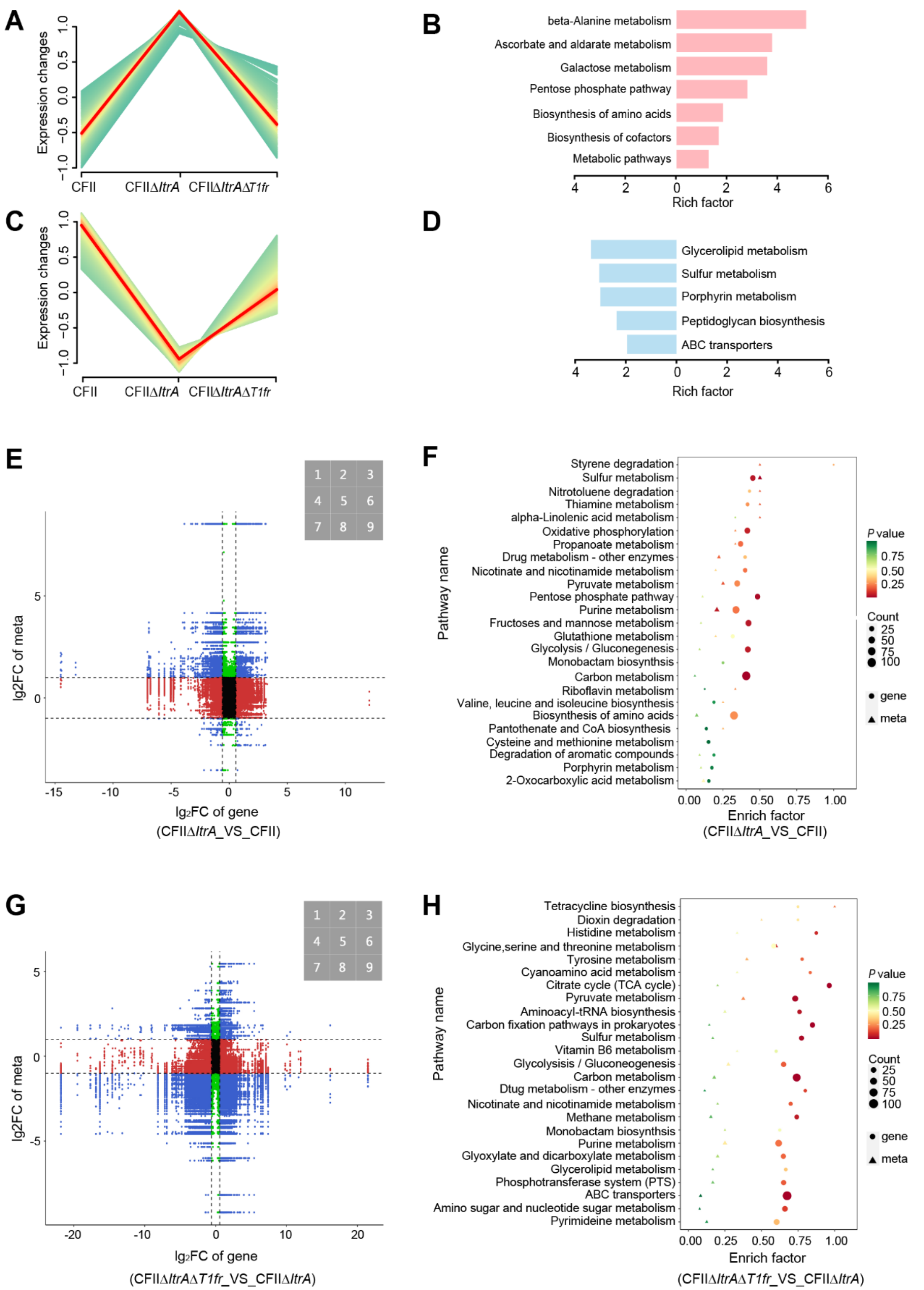

Fig. S10 *T1fr* regulated bacterial metabolic responses.

(A) The up-regulated gene cluster in the CFIIΔ*ltrA* strain compared to both CFII and

CFIIΔ*ltrA*Δ*T1fr* strains.

(B) The KEGG enrichment pathways of up-regulated gene clusters (Fig. S10A).

(C) The down-regulated gene cluster in the CFIIΔ*ltrA* strain compared to both CFII and CFIIΔ*ltrA*Δ*T1fr* strains.

(D) The KEGG enrichment pathways of down-regulated gene clusters (Fig. S10C).

(E) Nine-quadrant integration of omics data. Axes: $\log_2$(fold-change) of genes (x) and metabolites (y) in CFIIΔ*ltrA* *vs* CFII strains.

(F) Integrated omics (transcriptome and metabolome) analysis identified 25 significantly enriched pathways (FDR < 0.05) in the CFIIΔ*ltrA* *vs* CFII strains.

(G) Nine-quadrant integration of omics data. Axes: $\log_2$(fold-change) of genes (x) and metabolites (y) in CFIIΔ*ltrA*Δ*T1fr* *vs* CFIIΔ*ltrA* strains.

(H) Integrated omics (transcriptome and metabolome) analysis identified 25 significantly enriched pathways (FDR < 0.05) in the CFIIΔ*ltrA*Δ*T1fr* *vs* CFIIΔ*ltrA* strains.
