# Supplementary material for: A plasmid-encoded genetic switch orchestrates the bacterial virulence-fitness trade-off to drive antibiotic resistance dissemination
Source: mBio. 2026 Sep 16;17(10):e01899-26. doi: 10.1128/mbio.01899-26 (PMC13712751; doi:10.1128/mbio.01899-26)
Supplement: Supplementary file 2 [file mbio.01899-26-s0002.pdf]

Supplemental Tables (S1-S8)

A plasmid-encoded genetic switch balances virulence and fitness costs to drive global dissemination of antibiotic resistance

Hongcheng Wei [a, b, c, e #], Wenlan Yang [d #], Tiejun Zhang [a, b #], Wei Xu [a, b #], Ke Wu [a, b], Linghan Kong [a, b], Hao Li [a, b], Kailu Wang [a, b], Wenjun Yan [a, b], Ming Yang [a, b], Changyu Zhou [a, b], Songhua Li [d], Zhonghan Li [a], Changwei Lei [a, b *], Hongning Wang [a, b *]

[a] Laboratory of Bio-Resource and Eco-Environment of Ministry of Education, College of Life Sciences, Sichuan University, Chengdu, 610064, Sichuan Province, China

[b] Animal Disease Prevention and Food Safety Key Laboratory of Sichuan Province, Chengdu, 610064, China

[c] School of Laboratory Medicine, Chengdu Medical College, Chengdu, 610500, Sichuan Province, China

[d] Inner Mongolia Key Laboratory for Molecular Regulation of the Cell, College of Life Sciences, Inner Mongolia University, Hohhot 010021, China

[e] Chengdu Academy of Educational Sciences, 610036, Sichuan Province, China

[#] These authors contributed equally to this work

[*] Corresponding E-mail address: leichangwei@scu.edu.cn; hongningwang@scu.edu.cn.

Corresponding physical mailing address: 24 South Section 1, First Ring Road, Chengdu City, Sichuan Province, China This Supplementary data information contains:

- Supplementary Tables (**S1-S8**)

Table S1. Minimal inhibitory concentration (μg/mL) of antibiotics

**Strain**	Amp	SM	MEM	CFX	SXT	ERRY
EC600	<0.25	<0.25	<0.25	<0.25	<0.25	<0.25
CFII	≥256	<32	<64	<128	<0.5	<8
CFIIΔ*ltrA*	<256	<32	<64	<128	<1	<16
CFIIΔ*ltrA*Δ*T1fr*	<256	<32	<64	<128	<0.5	<16

Table S2. The information of plasmid carrying the *T1fr* gene (Plasmids obtained from the GenBank database or archived in our laboratory)

Accession	plasmid	*T1fr*	*ltrA*	Resistance genes carried on plasmids
CP054942.1	IncFII	YES	NO	blaTEM-1, rmtB1
CP047407.1	IncFII	YES	NO	blaTEM-1, rmtB1
CP047613.1	IncFII	YES	NO	mph(A), blaTEM-84
CP053725.1	IncFII	YES	NO	dfrA12, mph(A), fosA4, blaTEM-215, mcr-3.5
CP050754.1	IncFII, IncHI2, IncHI2A	YES	NO	sul1, qacEdelta1, arr-3, catB3, blaOXA-1, aac(6')-Ib-cr5, floR, sul2, aph(4)-Ia, aac(3)-IVa, aadA2, cmlA1, aadA1, qacL, sul3, aph(3')-Ia, dfrA12, sul1, bleO, oqxA, oqxB, catA2, blaCTX-M-55
CP050732.1	IncA/C2	YES	NO	floR, tet(A), aph(6)-Id, aph(3'')-Ib, sul2, catA2, aac(3)-IId, qnrS1, blaCTX-M-55, mcr-3.1, blaTEM-1
CP050727.1	IncA/C2	YES	NO	blaTEM-1, mcr-3.1, aac(3)-IId, qnrS1, blaCTX-M-55, catA2, sul2, aph(3'')-Ib, aph(6)-Id, tet(A), floR
CP050751.1	IncFII, IncHI2, IncHI2A	YES	NO	sul1, qacEdelta1, arr-3, catB3, blaOXA-1, aac(6')-Ib-cr5, floR, aph(6)-Id, aph(3'')-Ib, sul2, aph(4)-Ia, aac(3)-IVa, aadA2, cmlA1, aadA1, qacL, sul3, aph(3')-Ia, dfrA12, sul1, bleO, oqxA, oqxB, catA2, blaCTX-M-55
CP048372.1	IncFII	YES	NO	erm(B), mph(A), blaTEM-1, rmtB1
CP045953.1	IncA/C2	YES	NO	tet(A), aph(6)-Id, aph(3'')-Ib, sul2, aac(3)-IId, mcr-3.1, qnrS1
CP039562.1	IncA/C2	YES	NO	blaTEM-1, floR, tet(A), aph(6)-Id, aph(3'')-Ib, sul2, catA2, blaCTX-M-55, qnrS1, aac(3)-IId, mcr-3.1
MG014720.1	IncFII, IncHI2, IncHI2A	YES	NO	oqxB, oqxA, bleO, blaCTX-M-27, catA2, rmtB1, blaTEM-1, sul2, aph(4)-Ia, aac(3)-IVa, aac(6')-Ib-cr5, blaOXA-1, catB3, arr-3, qacEdelta1, sul1
CP032937.1	IncFII	YES	NO	mcr-3.1, aac(3)-IId, qnrS1, blaCTX-M-55, catA2
CP031655.1	IncFII	YES	NO	blaTEM-190, mph(A)
LR130554.1	IncFII	YES	NO	dfrA12, mph(A), fosA4, blaTEM-215
AP014634.1	IncA/C2	YES	NO	floR, tet(A), aph(6)-Id, aph(3'')-Ib, sul2, catA2, blaCTX-M-55, qnrS1, aac(3)-IId, mcr-3.1, blaTEM-1
MF554641.1	IncFIA, IncFIB(AP001918), IncFII, IncFII, IncI	YES	NO	blaTEM-1, rmtB1, blaCMY-42, mph(A), blaNDM-5, ble, sul1, qacEdelta1, aadA2, dfrA12, tet(B)
AP018147.1	IncFII	YES	NO	erm(B), mph(A), blaTEM-1, rmtB1, blaNDM-5, ble, sul1, qacEdelta1, aadA2, dfrA12
AP018139.1	IncFII	YES	NO	aac(3)-IIe, blaNDM-4, ble, sul1, qacEdelta1, aadA2, dfrA12, blaTEM-1, rmtB1
KY463220.1	IncFII	YES	NO	erm(B), mph(A), blaTEM-1, rmtB1, blaNDM-5, ble, sul1, qacEdelta1, aadA2, dfrA12
KX246267.1	IncFII	YES	NO	blaCTX-M-55, catA2
KY288024.1	IncFII	YES	NO	blaNDM, ble, sul1, qacEdelta1, aadA2, dfrA12
CP137407.1	IncFII, IncR	YES	NO	blaCTX-M-15, tet(A), dfrA1, qacEdelta1, sul1, mph(A), aac(3)-IIe, aac(6')-Ib-cr5, blaOXA-1, erm(B)
KX528699.1	Unknown	YES	NO	mcr-1.1
OR095746.1	IncFII	YES	NO	catA2, blaCTX-M, catA2, blaCTX-M-55
KR653209.1	IncFII, IncHI2, IncHI2A	YES	NO	tet(A), bleO, oqxA, oqxB, mph(A), aac(3)-IIe,aph(3'')-Ib,aph(6)-Id, blaTEM1, rmtB1, sul1, qacEdelta1, aadA2, dfrA12
CP091869.1	IncA/C2	YES	NO	floR, tet(A), aph(6)-Id, aph(3'')-Ib, sul2, blaTEM-1, mcr-3.1, aac(3)-IId, qnrS1, blaCTX-M-55, catA2
CP091867.1	IncA/C2	YES	NO	floR, tet(A), aph(6)-Id, aph(3'')-Ib, sul2, catA2, qnrS1, blaCTX-M-55, aac(3)-IId, mcr-3.1, blaTEM-1

LC056323.1	Unknown	YES	NO	
OQ658820.1	IncA/C2	YES	NO	floR, tet(A), aph(6)-Id, aph(3'')-Ib, sul2, catA2, blaCTX-M-55, qnrS1, blaTEM-1, aac(3)-IId, aac(3)-IId, mcr-3.1
KT002541.1	IncFII	YES	NO	blaTEM-1, rmtB1, blaCTX-M-98
CP064016.1	IncFII	YES	NO	mcr-3.5, qnrS1, blaCTX-M-55, catA2
CP064022.1	IncFII	YES	NO	aac(3)-IId, mcr-3.39, qnrS1, blaCTX-M-55, catA2
CP064010.1	IncFII	YES	NO	mcr-3.5, qnrS1, blaCTX-M-55, catA2
AP027965.1	IncFII	YES	NO	mcr-3.39, aac(3)-IId, qnrS1, blaCTX-M-55
CP107061.1	IncFII	YES	NO	erm(B), mph(A), sul1, qacEdelta1, aadA2, dfrA12, qnrS1, tet(A)
OP242230.1	Col440I, IncFII	YES	NO	mcr-3.4, mcr-3.4, aac(3)-IId, qnrS1, blaCTX-M-55, catA2
OP242229.1	IncFII	YES	NO	mcr-3, aac(3)-IId, qnrS1, blaCTX-M-55, catA2
LC744472.1	IncFII	YES	NO	erm(B), mph(A), blaTEM-1, rmtB1, blaNDM-5, ble, sul1, qacEdelta1, aadA2, dfrA12
LC744458.1	IncFII	YES	NO	aac(3)-IIe, blaNDM-4, ble, sul1, qacEdelta1, aadA2, dfrA12, blaTEM-1, rmtB1,
LC744453.1	IncFII	YES	NO	erm(B), mph(A), blaTEM-1, rmtB1, blaNDM-5, ble, sul1, qacEdelta1, aadA2, dfrA12
LC744451.1	IncFII	YES	NO	tet(A), blaTEM-1, rmtB1, blaNDM-5, ble, sul1, qacEdelta1, aadA2, dfrA12,
CP110995.1	IncFII	YES	NO	erm(B), mph(A), blaTEM-1, rmtB1, blaNDM-6, ble, sul1, qacEdelta1, aadA2, dfrA12
CP074044.1	IncFII	YES	NO	mph(A), blaCTX-M-32, dfrA8, qnrB19
CP073947.1	IncFII	YES	NO	blaCTX-M-32, sul3, dfrA8, qnrB19
CP073933.1	IncFII	YES	NO	blaCTX-M-32, mph(A), qnrB19
CP101380.1	IncA/C2	YES	NO	floR, tet(A), catA2, blaCTX-M-55, blaTEM-1, mcr-3.1
CP095666.1	IncFII	YES	NO	mph(A), blaTEM-1, blaNDM-5, ble, sul1, aadA2, dfrA12
CP095650.1	IncFII	YES	NO	erm(B), aadA2, qacEdelta1, sul1, ble, blaNDM-5, rmtB1, blaTEM-1, mph(A)
CP095628.1	IncFII	YES	NO	erm(B), mph(A), blaTEM, rmtB1, blaNDM-5, ble, sul1, qacEdelta1, aadA2, dfrA12
CP095608.1	IncFII	YES	NO	erm(B), mph(A), blaTEM-1, rmtB1, blaNDM-5, ble, sul1, qacEdelta1, aadA2, dfrA12
CP095153.1	IncFII	YES	NO	erm(B), mph(A), blaTEM-1
CP085624.1	IncFII	YES	NO	blaCTX-M-55, catA2
CP085059.1	IncFII(pCoo)	YES	NO	blaTEM-1, rmtB1, blaNDM-5, ble, sul1, qacEdelta1, aadA2, dfrA12
HG003695.1	IncFII	YES	NO	blaNDM-1, ble, sul1, qacEdelta1, aadA2, dfrA12, blaTEM-1, rmtB1
CP080371.1	IncFII	YES	NO	dfrA12, mph(A), fosA4, tet(X4), floR, blaTEM-215
CP080164.1	IncFII	YES	NO	dfrA12, mph(A), fosA4, tet(X4), floR, blaTEM-215
CP080156.1	IncFII	YES	NO	dfrA12, mph(A), fosA4, tet(X4), floR, blaTEM-215
CP080129.1	IncFII	YES	NO	tet(X4), floR, blaTEM-135
CP080134.1	IncFII	YES	NO	dfrA12, mph(A), fosA4, tet(X4), floR, blaTEM-215
CP080124.1	IncFII	YES	NO	aadA22, aph(3')-Ia, aph(6)-Id, aph(3'')-Ib, sul2, tet(X4), floR, blaTEM-215
OU015341.1	IncA/C2	YES	NO	floR, tet(A), aph(6)-Id, aph(3'')-Ib, sul2, aac(3)-IId, qnrS1, blaCTX-M-55, catA2, blaTEM-1
OU015324.1	IncA/C2	YES	NO	floR, tet(A), aph(6)-Id, aph(3'')-Ib, sul2, qnrS1, catA2, blaCTX-M-55, mcr-3.1, blaTEM-1

CP072807.1	IncFII	YES	NO	tet(X4), tet(X4), floR, blaTEM-215, aadA22, aph(3')-Ia, aph(6)-Id, aph(3'')-Ib, sul2
LC521838.1	IncFII	YES	NO	dfrA12, aadA2, qacEdelta1, sul1, ble, blaNDM-5, rmtB1, blaTEM-1, mph(A), erm(B)
LC521836.1	IncFII	YES	NO	dfrA12, aadA2, qacEdelta1, sul1, ble, blaNDM-5, rmtB1, blaTEM-1, mph(A), erm(B)
MT587865.1	IncFII	YES	NO	blaCTX-M-27, blaTEM-1, rmtB1
CP058357.1	IncFII	YES	NO	catA2, blaCTX-M-55
MT449722.1	IncFII	YES	NO	mcr-3.1, aac(3)-IId, qnrS1, blaCTX-M-55, catA2
MT449721.1	IncFII	YES	NO	catA2, blaCTX-M-55, qnrS1, mcr-3.5
MT449720.1	IncFII	YES	NO	mcr-3.1, aac(3)-IId, qnrS1, blaCTX-M-55, catA2
MN647789.1	IncA/C2	YES	NO	floR, tet(A), aph(6)-Id, aph(3'')-Ib, sul2, qnrS1, blaCTX-M-55, catA2, mcr-3.1, blaTEM-1
MN647788.1	IncA/C2	YES	NO	floR, tet(A), aph(6)-Id, aph(3'')-Ib, sul2, catA2, blaCTX-M-55, blaCTX-M-55, blaTEM-1, qnrS1, aac(3)-IId, mcr-3.1
CP138410.1	IncFII	YES	NO	tet(X4), blaTEM-215, floR
CP098233.1	IncFIA, IncFIB(AP001918), IncFIC(FII)	YES	NO	
CP011496.1	IncFIA, IncFIC(FII)	YES	NO	
CP099591.1	IncFIA, IncFIC(FII)	YES	NO	
CP058722.1	IncFII	YES	NO	dfrA12, mph(A), blaTEM-215
CP058712.1	IncFII	YES	NO	blaTEM-215, mph(A), dfrA12
CP068284.1	IncFIA, IncFIC(FII)	YES	NO	
CP068282.1	IncFIA, IncFIC(FII)	YES	NO	
CP068280.1	IncFIA, IncFIC(FII)	YES	NO	
CP057870.1	IncFIA, IncFIB(AP001918), IncFIC(FII)	YES	NO	
CP065471.1	Unknown	YES	NO	blaCMY
AP026457.1	IncFII(pSE11)	YES	NO	
CP091655.1	IncFII(pHN7A8)	YES	NO	
CP024864.1	IncFII(pSE11)	YES	NO	tet(A), blaTEM
AP027495.1	IncFIB(AP001918), IncFIC(FII)	YES	NO	
CP055983.1	IncFIC(FII)	YES	NO	
CP038858.1	IncR	YES	NO	
CP109877.1	Col8282, IncFII	YES	NO	
CP057180.1	IncFII(pCoo)	YES	NO	blaCMY-2
OK236218.1	IncFIB(AP001918),	YES	NO	dfrA17, aadA5, bleO, aadA2, dfrA12, sul3, qacL, aadA2, aac(3)-IId, oqxA, oqxB, floR, blaTEM-1

	IncFIC(FII)			
MT648288.1	IncFIB(AP001918), IncFIC(FII)	YES	NO	blaTEM-1, floR, dfrA17, aadA5, bleO, aadA2, dfrA12, sul3, qacL, aadA2, aac(3)-IId, oqxA, oqxB
OV049815.1	IncFII	YES	NO	dfrA12, aadA2, qacEdelta1, sul1, ble, blaNDM-5, rmtB1, blaTEM-1
CP057622.1	IncFIA, IncFIB(AP001918), IncFIC(FII)	YES	NO	
AP023236.1	IncFII	YES	YES	erm(B), mph(A), blaTEM-1, rmtB1, blaNDM-5, ble, sul1, qacEdelta1, aadA2, dfrA12
CP050367.1	IncFII	YES	YES	dfrA12, aadA2, aadA2, qacEdelta1, sul1, ble, blaNDM-5, rmtB1, blaTEM-1, mph(A), erm(B)
MN061455.1	IncFII	YES	YES	dfrA12, aadA2, qacEdelta1, sul1, ble, blaNDM-1, dfrA12, aadA2, qacEdelta1, sul1, ble, blaNDM-1, dfrA12, aadA2, qacEdelta1, sul1, ble, blaNDM-1, rmtB1, blaTEM-1
MN641485.1	IncFII	YES	YES	dfrA12, aadA2, qacEdelta1, sul1, ble, blaNDM-5, dfrA12, aadA2, qacEdelta1, sul1, ble, blaNDM-5, rmtB1, blaTEM-1
CP034254.1	IncFII	YES	YES	blaTEM-1, rmtB1, blaNDM-5, ble, sul1, qacEdelta1, aadA2, dfrA12
CP043863.1	IncFII	YES	YES	dfrA12, aadA2, qacEdelta1, sul1, ble, blaNDM-5, erm(B), mph(A)
CP041957.1	IncFII	YES	YES	blaNDM-5, ble, sul1, qacEdelta1, aadA2, dfrA12
CP041948.1	IncFII	YES	YES	blaNDM-5, ble, dfrA12
LC506720.1	IncFII	YES	YES	dfrA12, aadA2, qacEdelta1, sul1, erm(B), mph(A), blaTEM-1, rmtB1, blaNDM-5, ble
LC506719.1	IncFII	YES	YES	rmtB1, blaNDM-5, ble, sul1, qacEdelta1, aadA2, dfrA12
LC506718.1	IncFII	YES	YES	dfrA12, aadA2, qacEdelta1, sul1, erm(B), mph(A), blaTEM-1, rmtB1, blaNDM-5, ble
LC506717.1	IncFII	YES	YES	dfrA12, aadA2, qacEdelta1, sul1, erm(B), mph(A), blaTEM-1, rmtB1, blaNDM-5, ble
LC506716.1	IncFII	YES	YES	mph(A), blaTEM-1, rmtB1, blaNDM-5, ble
CP020517.1	IncFII	YES	YES	dfrA12, aadA2, qacEdelta1, sul1, ble, blaNDM-5, mph(A), erm(B), qnrS1
LS992188.1	IncFII	YES	YES	dfrA12, aadA2, qacEdelta1, sul1, ble, blaNDM-16b
MH213345.1	IncFII	YES	YES	blaTEM-1, rmtB1, blaNDM-16b, ble, sul1, qacEdelta1, aadA2, dfrA12
MG515250.1	IncFII	YES	YES	blaNDM-5, ble, sul1, qacEdelta1, aadA2, dfrA12, blaTEM-1, rmtB1
CP024430.1	IncFII, IncR	YES	YES	blaTEM-1, aph(6)-Id, aph(3'')-Ib, sul2, aph(6)-Id, aph(3'')-Ib, blaCTX-M-15, aadA2, dfrA12, catA2
CP026201.1	IncFII	YES	YES	erm(B), mph(A), blaTEM-1, rmtB1, blaNDM-5, ble, sul1, qacEdelta1, aadA2, dfrA12
CP024840.1	IncFII	YES	YES	dfrA12, aadA2, qacEdelta1, sul1, ble, blaNDM-5, rmtB1, blaTEM-1, mph(A), erm(B)
CP024836.1	IncFII	YES	YES	dfrA12, aadA2, qacEdelta1, sul1, ble, blaNDM-5, rmtB1, blaTEM-1, mph(A), erm(B)
CP023895.1	IncFII	YES	YES	mph(A), blaTEM-1, rmtB1, blaNDM-5, ble, sul1, qacEdelta1, aadA2, dfrA12
MF353156.1	IncFII	YES	YES	blaTEM-1, rmtB1, blaNDM-5, ble, sul1, qacEdelta1, aadA2, dfrA12, blaNDM-5, ble, sul1, qacEdelta1, aadA2, dfrA12
AP018144.1	IncFII	YES	YES	erm(B), mph(A), blaTEM-1, rmtB1, blaNDM-5, ble, sul1, qacEdelta1, aadA2, dfrA12
AP018138.1	IncFII	YES	YES	erm(B), mph(A), blaTEM-1, rmtB1, blaNDM-5, ble, sul1, qacEdelta1, aadA2, dfrA12
AP018136.1	IncFII	YES	YES	erm(B), mph(A), blaTEM-1, rmtB1
KY130431.1	IncFII	YES	YES	mph(A), blaTEM-1, rmtB1, blaNDM-5, ble, sul1, qacEdelta1, aadA2, dfrA12, erm(B)

CP135482.1	IncFIB(pQil), IncFII	YES	YES	erm(B), blaCTX-M-15, qacEdelta1, sul1, ble, blaNDM-5, rmtB1, blaTEM-1, mph(A)
CP130919.1	IncFII	YES	YES	blaTEM-1, rmtB1, blaNDM-5, ble, sul1, qacEdelta1, aadA2, dfrA12
CP015503.1	IncFII	YES	YES	catB, rmtF1, aac(6')-Ib, aadA2, dfrA12,
KT725789.1	IncFII	YES	YES	mph(A), blaTEM-1, rmtB1, blaNDM-5, ble, sul1, qacEdelta1, aadA2, dfrA12
KT725788.1	IncFII	YES	YES	erm(B), mph(A), blaTEM-1, rmtB1, blaNDM-5, ble, sul1, qacEdelta1, aadA2, dfrA12
OQ230786.1	IncFII	YES	YES	dfrA12, aadA2, qacEdelta1, sul1, ble, blaNDM-5, rmtB1, blaTEM-1
LN824138.1	IncFII	YES	YES	dfrA12, aadA2, aac(6')-Ib, rmtF1, catB
CP122472.1	IncFII	YES	YES	dfrA12, aadA2, qacEdelta1, sul1, erm(B), mph(A), blaTEM-1, qnrS1, catA1
LC744515.1	IncFII	YES	YES	erm(B), mph(A), blaTEM-1, rmtB1, blaNDM-5, ble, sul1, qacEdelta1, aadA2, dfrA12
LC744512.1	IncFII	YES	YES	erm(B), mph(A), blaTEM-1, rmtB1, blaNDM-5, ble, sul1, qacEdelta1, aadA2, dfrA12
LC744498.1	IncFII	YES	YES	erm(B), mph(A), dfrA12, aadA2, qacEdelta1, sul1, ble, blaNDM-5, rmtB1, blaTEM-1
LC744497.1	IncFII	YES	YES	erm(B), mph(A), blaTEM-1, rmtB1, blaNDM-5, ble, sul1, qacEdelta1, aadA2, dfrA12
LC744491.1	IncFII	YES	YES	erm(B), mph(A), blaTEM-1, rmtB1, blaNDM-5, ble, sul1, qacEdelta1, aadA2, dfrA12
LC744488.1	IncFII	YES	YES	erm(B), mph(A), blaTEM-1, rmtB1, blaNDM-5, ble, sul1, qacEdelta1, aadA2, dfrA12
LC744487.1	IncFII	YES	YES	erm(B), mph(A), blaTEM-1, rmtB1, blaNDM-5, ble, sul1, qacEdelta1, aadA2, dfrA12
LC744485.1	IncFII	YES	YES	blaTEM-1, rmtB1, blaNDM-5, ble, sul1, qacEdelta1, aadA2, dfrA12
LC744480.1	IncFII	YES	YES	erm(B), mph(A), blaTEM-1, rmtB1, blaNDM-5, ble, sul1, qacEdelta1, aadA2, dfrA12
LC744479.1	IncFIA, IncFIB(AP001918), IncFII, IncFII(pAMA1167-NDM-5)	YES	YES	aac(6')-Ib-cr5, blaCTX-M-5, dfrA12, aadA2, qacEdelta1, sul1, ble, blaNDM-5, rmtB1, blaTEM-1, tet(B), dfrA17, aadA5, qacEdelta1, sul1, aac(3)-IId
LC744477.1	IncFIA, IncFIB(AP001918), IncFII, IncFII(pAMA1167-NDM-5)	YES	YES	aac(6')-Ib-cr5, blaCTX-M-15, dfrA12, aadA2, qacEdelta1, sul1, ble, blaNDM-5, rmtB1, blaTEM-1, tet(B), dfrA17, aadA5, qacEdelta1, sul1, aac(3)-IId
LC744475.1	IncFII	YES	YES	erm(B), mph(A), blaTEM-1, rmtB1, blaNDM-5, ble, sul1, qacEdelta1, aadA2, dfrA12
LC744473.1	IncFII	YES	YES	erm(B), mph(A), blaTEM-1, rmtB1, blaNDM-5, ble, sul1, qacEdelta1, aadA2, dfrA12
LC744470.1	IncFII	YES	YES	erm(B), mph(A), blaTEM-1, rmtB1, blaNDM-5, ble, sul1, qacEdelta1, aadA2, dfrA12
LC744469.1	IncFIA, IncFIB(AP001918), IncFII, IncFII(pAMA1167-NDM-5)	YES	YES	aac(6')-Ib-cr5, blaCTX-M-15, dfrA12, aadA2, qacEdelta1, sul1, ble, blaNDM-5, rmtB1, blaTEM-1, blaOXA-1, blaTEM-1, mph(A), aph(6)-Id, aph(3'')-Ib, sul2, blaTEM-1, tet(B), dfrA17, aadA5, qacEdelta1, sul1, aac(3)-IId
LC744467.1	IncFII	YES	YES	erm(B), mph(A), blaTEM-1, rmtB1, blaNDM-5, ble, sul1, qacEdelta1, aadA2, dfrA12

LC744466.1	IncFII	YES	YES	blaTEM-1, rmtB1, blaNDM-5, ble, sul1, qacEdelta1, aadA2, dfrA12
LC744465.1	IncFII	YES	YES	erm(B), mph(A), blaTEM-1, rmtB1, blaNDM-5, ble, sul1, qacEdelta1, aadA2, dfrA12
LC744464.1	IncFII	YES	YES	blaTEM-1, rmtB1, blaNDM-5, ble, sul1, qacEdelta1, aadA2, dfrA12
LC744463.1	IncFII	YES	YES	blaTEM-1, rmtB1, blaNDM-5, ble, sul1, qacEdelta1, aadA2, dfrA12
LC744449.1	IncFII	YES	YES	erm(B), mph(A), blaTEM-1, rmtB1, blaNDM-1, ble, sul1, qacEdelta1, aadA2, dfrA12
LC744447.1	IncFII	YES	YES	rmtB1, blaTEM-1, blaNDM-5, ble, sul1, qacEdelta1, aadA2, dfrA12
LC744443.1	IncFII	YES	YES	erm(B), mph(A), blaTEM-1, rmtB1, blaNDM-5, ble, sul1, qacEdelta1, aadA2, dfrA12
LC744440.1	IncFII	YES	YES	erm(B), mph(A), blaTEM-1, rmtB1, blaNDM-5, ble, sul1, qacEdelta1, aadA2, dfrA12,
LC744439.1	IncFII	YES	YES	rmtB1, blaTEM-1, blaNDM-5, ble, sul1, qacEdelta1, dfrA12,
LC744436.1	IncFII	YES	YES	aac(6')-Ib-cr5, blaOXA-1, blaCTX-M-15, blaNDM-5, ble, sul1, qacEdelta1, aadA2, dfrA12
LC744434.1	IncFII	YES	YES	blaTEM-1, rmtB1, blaNDM-5, ble, sul1, qacEdelta1, aadA2, dfrA12
LC744433.1	IncFII	YES	YES	erm(B), mph(A), blaTEM-1, rmtB1, blaNDM-5, ble, sul1, qacEdelta1, aadA2, dfrA12
OM986070.1	IncFII	YES	YES	blaNDM-5, ble, sul1, qacEdelta1, aadA2, dfrA12,
CP096181.1	IncFII	YES	YES	blaTEM-1, rmtB1, blaNDM-5, ble, sul1, qacEdelta1, aadA2, dfrA12
CP096176.1	IncFII, IncR	YES	YES	blaTEM-1, rmtB1, blaNDM-5, ble, sul1, qacEdelta1, aadA2, dfrA12
CP095683.1	IncFII	YES	YES	erm(B), mph(A), blaTEM-1, rmtB1, blaNDM-5, ble, sul1, qacEdelta1, aadA2, dfrA12
CP101617.1	IncFII	YES	YES	blaTEM-1, rmtB1, blaNDM-5, ble, sul1, qacEdelta1, aadA2, dfrA12
CP095681.1	IncFII	YES	YES	erm(B), mph(A), blaTEM-1, rmtB1, blaNDM-5, ble, sul1, qacEdelta1, aadA2, dfrA12
CP095670.1	IncFII	YES	YES	erm(B), mph(A), blaTEM-1, rmtB1, blaNDM-5, ble, sul1, qacEdelta1, aadA2, dfrA12
CP095669.1	IncFII	YES	YES	erm(B), mph(A), blaTEM-1, rmtB1, blaNDM-5, ble, sul1, qacEdelta1, aadA2, dfrA12
CP095664.1	IncFII	YES	YES	blaTEM-1, rmtB1, blaNDM-5, ble, sul1, qacEdelta1, aadA2, dfrA12
CP095663.1	IncFII	YES	YES	erm(B), mph(A), blaTEM, rmtB1, blaNDM-5, ble, sul1, qacEdelta1, aadA2, dfrA12
CP095656.1	IncFII(29)	YES	YES	blaTEM-1, rmtB1, blaNDM-5, ble, sul1, qacEdelta1, aadA2, dfrA12
CP095655.1	IncFII	YES	YES	mph(A), blaTEM-1, rmtB1, blaNDM-5, ble, sul1, qacEdelta1, aadA2, dfrA12
CP095648.1	IncFII	YES	YES	erm(B), mph(A), blaTEM-1, rmtB1, blaNDM-5, ble, sul1, qacEdelta1, aadA2, dfrA12
CP095632.1	IncFII	YES	YES	erm(B), mph(A), blaTEM-1, rmtB1, blaNDM-5, ble, aadA2, dfrA12
CP095626.1	IncFII	YES	YES	dfrA12, aadA2, qacE, ble, blaNDM-5, rmtB1, blaTEM-1, mph(A)
CP095625.1	IncFII	YES	YES	blaTEM-1, rmtB1, blaNDM-5, ble, sul1, qacEdelta1, aadA2, dfrA12
CP095624.1	IncFII	YES	YES	mph(A), blaTEM-1, rmtB1, blaNDM-5, ble, sul1, qacEdelta1, aadA2, dfrA12
CP095622.1	IncFII	YES	YES	erm(B), mph(A), blaTEM-1, rmtB1, blaNDM-5, ble, sul1, qacEdelta1, aadA2, dfrA12
CP095621.1	IncFII	YES	YES	erm(B), mph(A), blaTEM-1, rmtB1, blaNDM-5, ble, sul1, qacEdelta1, aadA2, dfrA12
CP095619.1	IncFII	YES	YES	mph(A), blaNDM, qacEdelta1, aadA2, dfrA12
CP095618.1	IncFII	YES	YES	erm(B), mph(A), blaTEM-1, rmtB1, blaNDM-5, ble, sul1, qacEdelta1, aadA2, dfrA12
CP095616.1	IncFII	YES	YES	rmtB1, blaTEM-1, blaNDM-5, ble, sul1, qacEdelta1, aadA2, dfrA12
CP095615.1	IncFII	YES	YES	mph(A), blaNDM-5, ble, sul1, qacEdelta1, aadA2, dfrA12

CP095611.1	IncFII	YES	YES	erm(B), mph(A), blaTEM-1, rmtB1, blaNDM-5, ble, sul1, qacEdelta1, aadA2, dfrA12
CP095610.1	IncFII	YES	YES	erm(B), mph(A), dfrA12, aadA2, qacEdelta1, sul1, ble, blaNDM-5, rmtB1
CP095606.1	IncFII	YES	YES	erm(B), mph(A), rmtB1, blaNDM-5, ble, sul1, qacEdelta1, aadA2, dfrA12
CP095605.1	IncFII	YES	YES	erm(B), blaTEM-1, rmtB1, blaNDM-5, ble, sul1, qacE, aadA2, dfrA12
CP095601.1	IncFII	YES	YES	erm(B), rmtB1, blaTEM-1, mph(A), blaNDM-5, ble, qacEdelta1, aadA2, dfrA12
CP095595.1	IncFII	YES	YES	erm(B), rmtB1, blaTEM-1, mph(A), blaNDM-5, ble, sul1, qacEdelta1, aadA2, dfrA12
CP095594.1	IncFII	YES	YES	blaNDM-5, ble, sul1, qacEdelta1, aadA2, dfrA12
CP095588.1	IncA/C2, IncFII	YES	YES	sul2, blaCMY-16, dfrA1, aadA5, qacEdelta1, sul1, ble, blaNDM-5, dfrA12, aadA2, qacEdelta1, sul1, armA, msr(E), mph(E), blaOXA-1, aac(6')-Ib-cr5
CP095580.1	IncFII, IncFII(K), IncR	YES	YES	catA1, blaOXA-1, aac(6')-Ib-cr5, erm(B), mph(A), blaTEM-1, qnrS1, sul1, qacEdelta1, dfrA12, aadA2, qacEdelta1, sul1, ble, blaNDM-5, rmtB1, blaTEM-1, blaCTX-M-15
CP095579.1	IncFII	YES	YES	rmtB1, blaNDM-5, ble, sul1, qacEdelta1, aadA2, dfrA12
CP095576.1	IncFII	YES	YES	erm(B), mph(A), blaTEM-1, blaNDM-5, ble, sul1, qacEdelta1, aadA2, dfrA12
CP095574.1	IncFII	YES	YES	erm(B), mph(A), blaTEM-1, rmtB1, blaNDM-5, ble, sul1, qacEdelta1, aadA2, dfrA12
CP095569.1	IncFII	YES	YES	blaNDM, sul1, qacEdelta1, dfrA12
AP023434.1	IncFII	YES	YES	erm(B), mph(A), blaTEM-1, rmtB1, blaNDM-5, ble, sul1, qacEdelta1, aadA2, dfrA12
MT621569.1	IncFII	YES	YES	dfrA12, aadA2, qacEdelta1, sul1, ble, blaNDM-5, rmtB1, blaTEM-1
MZ892875.1	IncFII	YES	YES	erm(B), mph(A), blaTEM-1, rmtB1, blaNDM-5, ble, sul1, qacEdelta1, aadA2, dfrA12
CP080175.1	IncFII	YES	YES	blaTEM-215, floR, tet(X4), tet(X4), fosA4, mph(A), dfrA12
CP074117.1	Unknown	YES	YES	dfrA12, aadA2, qacEdelta1, sul1, ble, blaNDM-5
CP058942.1	IncFII	YES	YES	erm(B), mph(A), blaTEM-1, rmtB1, blaNDM-5, ble, sul1, aadA2, dfrA12
AP023231.2	IncFII	YES	YES	blaNDM-5, ble, sul1, qacEdelta1, aadA2, dfrA12
AP023220.2	IncFII	YES	YES	erm(B), mph(A), blaTEM-1, rmtB1, blaNDM-5, ble, sul1, qacEdelta1, aadA2, dfrA12
CP063481.1	IncFII(pHN7A8)	YES	YES	sul1, qacEdelta1, aadA2, dfrA12, blaTEM-1, rmtB1, blaNDM-5, ble
CP048012.1	IncFIIIncR	YES	YES	blaTEM-1, rmtB1, blaNDM-5, ble, sul1, qacEdelta1, aadA2, dfrA12
JQ364967.1	IncFII	YES	YES	dfrA12, aadA2, qacEdelta1, sul1, ble, blaNDM-1, aac(3)-IIe, blaOXA-1, aac(6')-Ib-cr5
CP054171.1	IncFII	YES	YES	erm(B), qacEdelta1, sul1, ble, blaNDM-5, rmtB1, blaTEM-1, mph(A)
AP023454.1	IncFII	YES	YES	erm(B), mph(A), blaTEM-1, rmtB1, blaNDM-5, ble, sul1, qacEdelta1, aadA2, dfrA12
CP058801.1	IncFII	YES	YES	erm(B), mph(A), blaTEM-1, rmtB1, blaNDM-5, ble, sul1, qacEdelta1, aadA2, dfrA12
CP053768.1	IncFII	YES	YES	erm(B), mph(A), rmtB1, blaNDM-5, ble, sul1, qacEdelta1, aadA2, dfrA12
CP095586.1	IncFII	YES	YES	erm(B), rmtB1, blaTEM-1, mph(A), blaNDM, ble, sul1, qacEdelta1, aadA2, dfrA12
CP095623.1	IncFII	YES	YES	blaNDM-5, ble, sul1, qacEdelta1, aadA2, dfrA12, blaTEM, mph(A)
CP095582.1	IncFII	YES	YES	mph(A), ble, sul1, qacEdelta1, aadA2, dfrA12

Table S3. Bacterial strains involved in this study

Name	Description	Source
EC51	Multi-drug resistant *Escherichia coli* including plasmid pEC51 isolated from poultry farm	This study (GCA_029087925.1)
EC600	Rifampicin-resistant *Escherichia coli*	Invitrogen
CFII	pEC51-carrying EC600 strain	This study
CFIIΔ*ltrA*	EC600 *ltrA* deletion mutant	This study
CFIIΔ*ltrA*Δ*T1fr*	EC600 *ltrA* and *T1fr* deletion mutant	This study
LF82	Adherent invasive *Escherichia coli* (AIEC)	BioSci
BL21-DE3	Commercial *Escherichia coli* for protein expression	Invitrogen
DH5a	Commercial *Escherichia coli* for plasmid cloning	Sangon

Table S4. Plasmids used in this study

Name	Description	Source
pEC51	A plasmid containing both ItrA and T1fr genes isolated and identified by our laboratory.	This study (Accession no. OQ230786.1)
pKD46	Encodes λ recombinase; survives at 30°C; carries tetracycline resistance gene.	MIAOLING
pKD3	Carries FRT flanking chloramphenicol cassette with downstream RBS.	MIAOLING
pCP20	Encodes FLP recombinase; survives at 30°C; carries tetracycline resistance gene.	MIAOLING
pUC19	Commercial plasmid vector	MIAOLING
pEC51Δ*ltrA*	pEC51 *ltrA* deletion mutant	This study
pEC51Δ*ltrA*Δ*T1fr*	pEC51 *ltrA* and *T1fr* deletion mutant	This study
pUC19+*ltrA*	pUC19 carrying *ltrA* with mCherry gene	This study
pUC19+*T1fr*	pUC19 carrying *T1fr* with mCherry gene	This study
pET-28a	Commercial plasmid vector	Sangon
pET-28a+*ltrA*	pUC19 carrying *ltrA* with T7 promoter	This study
pET-28a+*T1fr*	pUC19 carrying *T1fr* with T7 promoter	This study

Table S5. Primers used in this study

Primers	Sequence
IncFII-F	CTGATCGTTTAAGGAATTTT
IncFII-R	CACACCATCCTGCACTTA
bla_{NDM}-F	GGTTTGGCGATCTGGTTTTC
bla_{NDM}-R	CGGAATGGCTCATCACGATC
16SrRNA-F	AGTTTGATC(A/C)TGGCTCAG
16SrRNA-R	GGACTAC(C/T/A)AGGGTATCTAAT
ltrA-F	CGCATCCTCACCCGCTCATTCT
ltrA-R	TGGGCGATACTTTCTGGCTCAT
pKD46-F	TTTGAGTTGTGGGTATCTGT
pKD46-R	CTTGTATTATGGGTAGTTTCC
pCP20-F	TACGCTGACTTGACGGGACG
pCP20-R	GTGAGGGAGCCACGGTTGA
ltrA-ko-F	GTCTTTAATTGCATGAAAAGCTGTAAAGCAGTCAAAAGGATATCAG CGAGTGTGTAGGCTGGAGCTGCTTCG
ltrA-ko-R	CGCCCCCTAAGAACGGAGCGTGCGAGTTTCCCCGCACTCCGCTC AAGCCTCATATGAATATCCTCCTTAG
ltrA-ko(check)-F1	CGCCCTGACCCTCTGTATGT
ltrA-ko(check)-R1	TTTCGTCTCAGCCAATCCCT
ltrA-ko(check)-F2	AACAGGGACACCAGGATTTATTT
ltrA-ko(check)-R2	TTATCTGCTGCTCTGATTCTGACTA
Chl-F	GACGGTGAGCTGGTGATATG
Chl-R	TCACCGTAACACGCCACATC
pUC19-F	GGCAACTATGGATGAACG
pUC19-R	GCAAGCAGCAGATTACGC
fimA-F	GCACAGGAAGGAGCAACCA
fimA-R	GTCGCATCCGCATTAGCAG
fimB-F	CGGGTTGTCACTGGAGATT
fimB-R	TATTGCGATGCCCAAGATA
fimC-F	GCCGATGGTGTAAAGGAT
fimC-R	CCGTCAGGTAATAGGGTGT
fimD-F	ACGATACGGTGGTGCTTGT
fimD-R	CGGCTTATTATTGTGGGTCA
fimE-F	GCAGGCGGTTTGTTACGGG
fimE-R	TCCACGGCTTCACGCTCAT
fimF-F	CGACGACTCCTGTTGTTCC
fimF-R	AGGTTGGCATTGTGGCTAT
fimG-F	TCGGCGATCTTTATTCTTT
fimG-R	GTTGCCACTGTCATCCTGT
fimH-F	TGATGGGCTGGTCGGTAAA

fimH-R	CGCTGGTGGTAGGAAATGG
fimI-F	GCTGCCAATGTTTGCTCTG
fimI-R	CCTACACGTTCACTCACCACC
fimS-ON-F	GCGGAGGTGATGTGAAATTA
fimS-OFF-F	CTCCAAAAACCACCTCAT
fimS-ON-450-F	CTCATAGAGGAAAGCATCGC
fimS-ON-250-F	CATGAGGTGGTTTTTGGAG
fimS-OFF-118/285-F	CTCCAAAAACCACCTCATG
fimS-external-R	AGAGCCGACAGAACAACGAT
fimS-internal-OFF-R	CTCATAGAGGAAAGCATCGC
T1fr-F	TGTCCGCAGCAACCTTAC
T1fr-R	ATTTGTTTCCTGGTCACTCA
T1fr-ko-F	AAAGCTTTTATAGTGAGGGACTTCAGAAATACCCTAGAAAAGGAAC TGTTCATATGAATATCCTCCTTAG
T1fr-ko-R	CCGCCAGGCCCACTCTCCGGTCGTGGCCGCCTTAAGGACGGTTC CGTTCTTGTGTAGGCTGGAGCTGCTTCG
T1fr-ko(check)-F1	GGGATTAGGTCAGGACAGAT
T1fr-ko(check)-R1	TCAACAGGGACACCAGGATT
T1fr-ko(check)-F2	GGACAGATAGCCTGAAAGC
T1fr-ko(check)-R2	CAACTGACTGAAATGCCTC
GW576_23915-F	GAGTACCCGAAATATCCACG
GW576_23915-R	GGCACGCTTAATCTGACG
GW576_23915-ko-F	GAAACTCAACAGATGATGGGGGCTGAAAACCAGAGAGAGGAGAA AGCAGGCATATGAATATCCTCCTTAG
GW576_23915-ko-R	TCTCCATTAACAAGATTGCAACAATCAGGAGGGATATTCATCACATC CGGTGTGTAGGCTGGAGCTGCTTCG
GW576_23915-ko(check)-F1	CATAGACAGCCACAACCG
GW576_23915-ko(check)-R1	CAACAGGGACACCAGGAT
GW576_23915-ko(check)-F2	GGGCATAGACAGCCACAACC
GW576_23915-ko(check)-R2	TCAACAGGGACACCAGGATT

Table S6. A summary of antibodies and reagents information used in this study

Reagent or resource	Source	Identifier
**Antibodies**		
Mouse anti His-Tag mAb	Abclonal	Cat#: AE003
horseradish peroxidase (HRP)-conjugated anti-rabbit IgG antibody	Abcam	Cat#: ab205718
**chemicals, peptides, and recombinant proteins**		
Dulbecco's MEM (DMEM) high glucose	Thermo Fisher	Cat#: C11995500BT
Penicillin-Streptomycin solution (100×)	Gibco	Cat#: 15140-122
Fetal bovine serum	Gibco	Cat#: 10091148
TRIzol™ reagent	Thermo Fisher	Cat#: 16096020
RNase-free Tris-HCl buffer (1 M, pH 9.0)	TIANDZ	Cat#: 80933
Protease inhibitor cocktail	Sigma-Aldrich	Cat#: P8340
Anti-Flag M2 Affinity Gel	Sigma-Aldrich	Cat#: A2220
3 × Flag peptide	Sigma-Aldrich	Cat#: F4799
anti-FLAG® M2 magnetic beads	sigma	Cat#: M8823
5 × Hi-Density TBE sample buffer	Invitrogen	Cat#: LC6678
6% TBE gel	Invitrogen	Cat#: EC6865BOX
RNase inhibitor	Thermo Fisher	Cat#: EO0381
NuPAGE™ 4-12% Bis-Tris Gel	Invitrogen	Cat#: NP0335BOX
NuPAGE™ LDS sample buffer (4×)	Invitrogen	Cat#: NP0007
DIG RNA labeling mix	Roche	Cat#: 11277073910
Tetracycline	Macklin	Cat#: 60-54-8
Chloramphenicol	Macklin	Cat#: 56-75-7
Meropenem	Macklin	Cat#: 96036-03-2
Mouse Interleukin 6 (IL-6) ELISA Kit	MEIMIAN	Cat#: MM-1011M1
Mouse Interleukin 8 (IL-8) ELISA Kit	MEIMIAN	Cat#: MM-0123M1

Mouse Tumor necrosis factor α (TNF-α) ELISA Kit	MEIMIAN	Cat#: MM-47831M1
RevertAid^{TM} first strand cDNA synthesis kit	Thermo Fisher	Cat#: K1622
SYBR Permix Ex Taq	Takara	Cat#: RR420A

Table S7. Oligonucleotide sequence used in this study

**Name**	**Sequence (5`-3`)**
T1fr-promoter-EMSA (5`Biotin)	GGGATTAGGTCAGGACAGATAGCCTGAAAGCTTTTATAGTGAGGGACTTCAGAAATACCCTAGAAAAGGAACTGTT
T1fr-Mut-promoter-EMSA (5`Biotin)	GGGATTAGGTCAGGACAGATAGCCTGAAAGTGAGGGACTTCAGAAATACCCTAGAAAAGGAACTGTT
fimS-EMSA (5`Biotin)	TGCAATATAAACATCTATAAATAAAGATAACAATAGAATATTAAGCCAACAAATAAACTGAAAAAGTTTGTCCGCGATGCTTTCCACTATGAGTCAAAA
fimS-Mut-EMSA (5`Biotin)	TGCAATATAAACATCTATAGAATATTAAGCCAACAAATAAACTGAAAAAGTTTGTCCGCGATGCTTTCCACTATGAGTCAAAA
fimS(1)-EMSA (5`Biotin)	CTATTGTAAATTAATTTCACATCACCTCCGCTATATGTAAAGCTAACGTTTCTGTGGCTCGACGCATCTTCCTCATTCTTCTCTCCAAAAACCACCTCA
fimS(2)-EMSA (5`Biotin)	AACTGTCCATATCATAAATAAGTTACGTATTTTTTCTCAAGCATAAAAATATTAAAAAACGACAAAAAGCATCTAACTGTTTGATATGTAAATTATTT

Table S8. DNA-protein binding of the *fimA* promoter (*fimS*) predicted by SAResNet

Name	Sequence	Score
fimA-1	TTGGGGCCAAACTGTCCATATCATAAATAAGTTACGTATTTTTTCTCAAGCATAAAAATATTAAAAAACGACAAAAAGCATCTAACTGTTTGATATGTAAA	0.480
fimA-2	ATCTAACTGTTTGATATGTAAATTATTTCTATTGTAAATTAATTTCACATCACCTCCGCTATATGTAAAGCTAACGTTTCTGTGGCTCGACGCATCTTCCT	0.685
fimA-3	CGTTTCTGTGGCTCGACGCATCTTCCTCATTCTTCTCTCCAAAAACCACCTCATGCAATATAAACATCTATAAATAAAGATAACAATAGAATATTAAGCCA	0.892
fimA-4	TAAACATCTATAAATAAAGATAACAATAGAATATTAAGCCAACAAATAAACTGAAAAAGTTTGTCCGCGATGCTTTCCTCTATGAGTCAAAATGGCCCCAA	0.769
fimA-5	AAATTAATTTCACATCACCTCCGCTATATGTAAAGCTAACGTTTCTGTGGCTCGACGCATCTTCCTCATTCTTCTCTCCAAAAACCACCTCATGCAATATA	0.547
fimA-6	ACCTCCGCTATATGTAAAGCTAACGTTTCTGTGGCTCGACGCATCTTCCTCATTCTTCTCTCCAAAAACCACCTCATGCAATATAAACATCTATAAATAAA	0.856
fimA-7	TGTAAAGCTAACGTTTCTGTGGCTCGACGCATCTTCCTCATTCTTCTCTCCAAAAACCACCTCATGCAATATAAACATCTATAAATAAAGATAACAATAGA	0.931
fimA-8	CTAACGTTTCTGTGGCTCGACGCATCTTCCTCATTCTTCTCTCCAAAAACCACCTCATGCAATATAAACATCTATAAATAAAGATAACAATAGAATATTAA	0.930
fimA-9	GCTATATGTAAAGCTAACGTTTCTGTGGCTCGACGCATCTTCCTCATTCTTCTCTCCAAAAACCACCTCATGCAATATAAACATCTATAAATAAAGATAAC	0.938
fimA-10	TCCGCTATATGTAAAGCTAACGTTTCTGTGGCTCGACGCATCTTCCTCATTCTTCTCTCCAAAAACCACCTCATGCAATATAAACATCTATAAATAAAGAT	0.879
fimA-11	ATATGTAAAGCTAACGTTTCTGTGGCTCGACGCATCTTCCTCATTCTTCTCTCCAAAAACCACCTCATGCAATATAAACATCTATAAATAAAGATAACAAT	0.936
fimA-12	CTATATGTAAAGCTAACGTTTCTGTGGCTCGACGCATCTTCCTCATTCTTCTCTCCAAAAACCACCTCATGCAATATAAACATCTATAAATAAAGATAACA	0.938
fimA-13	TATATGTAAAGCTAACGTTTCTGTGGCTCGACGCATCTTCCTCATTCTTCTCTCCAAAAACCACCTCATGCAATATAAACATCTATAAATAAAGATAACAA	0.950

fimA-14	CCGCTATATGTAAAGCTAACGTTTCTGTGGCTCGACGCATCTTCCTCATTCTTCTCTCCAAAAACCACCTCATGCAATATAAACATCTATAAATAAAGATA	0.902
fimA-15	CGCTATATGTAAAGCTAACGTTTCTGTGGCTCGACGCATCTTCCTCATTCTTCTCTCCAAAAACCACCTCATGCAATATAAACATCTATAAATAAAGATAA	0.914
